# A Single-Chain-Based Hexavalent CD27 Agonist Enhances T Cell Activation and Induces Anti-Tumor Immunity

**DOI:** 10.3389/fonc.2018.00387

**Published:** 2018-09-19

**Authors:** Meinolf Thiemann, David M. Richards, Karl Heinonen, Michael Kluge, Viola Marschall, Christian Merz, Mauricio Redondo Müller, Tim Schnyder, Julian P. Sefrin, Jaromir Sykora, Harald Fricke, Christian Gieffers, Oliver Hill

**Affiliations:** Apogenix AG, Heidelberg, Germany

**Keywords:** single-chain CD27L, scCD27L-RBD, CD27, agonist, TNFSF, TNFRSF7, CD70, HERA

## Abstract

Tumor necrosis factor receptor superfamily member 7 (TNFRSF7, CD27), expressed primarily by T cells, and its ligand CD27L (TNFSF7, CD70) provide co-stimulatory signals that boost T cell activation, differentiation, and survival. Agonistic stimulation of CD27 is therefore a promising therapeutic concept in immuno-oncology intended to boost and sustain T cell driven anti-tumor responses. Endogenous TNFSF/TNFRSF-based signal transmission is a structurally well-defined event that takes place during cell-to-cell-based contacts. It is well-established that the trimeric-trivalent TNFSF-receptor binding domain (TNFSF-RBD) exposed by the conducting cell and the resulting multi-trimer-based receptor clustering on the receiving cell are essential for agonistic signaling. Therefore, we have developed HERA-CD27L, a novel *he*xavalent TNF *r*eceptor *a*gonist (HERA) targeting CD27 and mimicking the natural signaling concept. HERA-CD27L is composed of a trivalent but single-chain CD27L-receptor-binding-domain (scCD27L-RBD) fused to an IgG1 derived silenced Fc-domain serving as dimerization scaffold. The hexavalent agonist significantly boosted antigen-specific T cell responses while having no effect on non-specific T cells and was superior over stabilized recombinant trivalent CD27L. In addition, HERA-CD27L demonstrated potent single-agent anti-tumor efficacy in two different syngeneic tumor models, MC38-CEA and CT26wt. Furthermore, the combination of HERA-CD27L and an anti-PD-1 antibody showed additive anti-tumor effects highlighting the importance of both T cell activation and checkpoint inhibition in anti-tumor immunity. In this manuscript, we describe the development of HERA-CD27L, a true CD27 agonist with a clearly defined forward-signaling mechanism of action.

## Introduction

Strategies to enhance anti-tumor immune responses are among the most promising new developments in oncology due to important characteristics of the adaptive immune system; including, antigen-specificity, adaptability, and ability to generate long-lasting memory (1). The diverse functions of the immune system are orchestrated by a complex and delicately balanced interplay of stimulatory and inhibitory signals. Many key regulators of this symphony are ligands belonging to the tumor necrosis factor superfamily (TNFSF) and their cognate receptors, collectively called the TNF receptor superfamily (TNFRSF) (2–4). The TNFSF consists of 19 structurally related ligands, each binding to one or more of the 29 members of the TNFRSF (2, 5). TNFRSF receptors are expressed by a wide variety of immune cells including T cells and antigen-presenting cell (APC) populations, such as dendritic cells and macrophages, as well as by tumor cells themselves (2, 3). This diverse expression pattern highlights the critical role that they play in many locations and phases of the anti-tumor immune response. CD27 (TNFRSF7), found on T cells and some natural killer (NK) cell populations, is expressed at particularly high levels by naïve T cells and regulatory T (Treg) cells. CD27 signaling, induced by its ligand CD70 (TNFSF7, CD27L), plays an important role in regulating immune responses by providing co-stimulatory signals to boost T cell activation, differentiation, survival and memory formation (3, 6, 7). CD27 is therefore an especially important target for immunotherapy.

Various strategies to induce CD27 signaling are currently under investigation and they can be broadly grouped into CD27L-based or agonistic antibody-based approaches (4, 8–11). Due to their unique receptor clustering patterns, generating productive downstream signals from TNFRSF receptors is dependent on agonistic compounds having a very precise exposure and structure. TNFSF ligands naturally assemble on the cell surface as homo-trimers containing three receptor binding sites. The interaction of these trimeric TNFSF ligands with their corresponding receptors, expressed on the surface of other cells, leads to very precise receptor clustering followed by intracellular signal transduction (2, 8, 11–13). The trimeric structure of the TNFSF ligands and the resulting receptor clusters are prerequisites for productive signal transmission into the cell. Due to these specific requirements, monovalent and bivalent approaches have generally proven to be minimally effective *in vivo* (8, 11–13).

We have previously described the construction and biological activity of a *he*xavalent TNF *r*eceptor *a*gonist (HERA) molecule targeting TRAIL receptors (14). We showed that the unique molecular layout and binding mode of HERA-TRAIL/APG350, a prototype death receptor agonist, conferred potent biological activity that was superior to conventional agonistic antibodies and independent of additional crosslinking via Fcγ receptors (FcγR) (11, 14). The underlying HERA structure was shown to overcome the significant limitations of bivalent antibody-based approaches because it mimics the natural trimeric ligands, thus inducing optimal trimeric assembly of the TNFRSF receptors. This approach has been validated for a member of the cytotoxic arm of the TNFSF. In this manuscript, we describe the creation of a novel co-stimulatory HERA molecule that is able to target CD27 and furthermore use HERA-CD27L to demonstrate the powerful immuno-stimulatory and anti-tumor activity of this approach.

## Materials and methods

### Construction, expression, and purification of hexavalent HERA-CD27L and trimeric CD27L

To engineer a hexavalent CD27 agonist, we designed a single-polypeptide chain with three copies of a CD27L (CD70) protomer sub-sequence. In detail, three copies from human CD27L comprising amino acids E51-P193 were interconnected with two glycine-serine based linkers 8 aa in length. The resulting trivalent single chain-CD27L-receptor-binding-domain (scCD27L-RBD) was fused to the Fc-part of a human IgG1-mutein, which is deficient for Fcγ receptor binding, to create an Fc-silenced hexavalent scCD27L-RBD dimer. A stabilized homotrimeric CD27L-receptor-binding domain was obtained by fusing a bacteriophage derived trimerization domain (Enterobacteria phage RB69 fibritin amino acids G455-A480) C-terminal to the human CD27L (E51-P193) subsequence employing a glycine/serine-based linker-element. In addition, both CD27L constructs contain a C-terminal Strep-Tag II for purification purposes. Secretory pathway-based expression was achieved by adding an appropriate signal-peptide to their N-Terminus. Codon optimized synthetic genes were obtained from GeneWiz, re-cloned into proprietary expression vectors and transfected into suspension-adapted Chinese Hamster Ovary cells (CHO-S cells, Invitrogen).

HERA-CD27L content of the supernatants was monitored by ELISA during the selection process and the best-expressing cell pools were subsequently expanded for protein production. For expression and purification, high titer cell pools were expanded in a WAVE bioreactor (GE Healthcare) at 37°C with 7% CO_2_ for seven to 12 days in chemically defined medium (PowerCHO2-CD, Lonza) with two feeds (PowerFeed A, Lonza). Cells were harvested when cell viability dropped below 70% and the supernatant was clarified by centrifugation and filtration prior to purification. The hexavalent HERA-CD27L and the trimeric CD27L were purified by a two-step process combining Streptactin affinity purification (AFC) followed by preparative size-exclusion chromatography (SEC) both performed under physiological buffer conditions in PBS at pH 7.4. Purity and integrity of the protein was confirmed by analytical SEC as well as denaturing and non-denaturing SDS-PAGE.

### Construction, expression, and purification of an anti-human CD27 antibody (1F5)

The V_L_ and V_H_ sequences for the humanized monoclonal anti-CD27 antibody 1F5 were derived from US patent application US20120213771A1. A full length IgG1 format was generated by fusing appropriate constant light-chain and heavy chain domains to the V_L_ and V_H_ chain. The resulting full format antibody was produced in CHO-S cells employing the same methods as described for the CD27L-constructs. Finally, high molecular weight species of the AFC purified antibody were removed by SEC. The specificity and activity of antibody preparations were determined as described before (15).

### CD27 signaling and biological activity reporter assay

We evaluated CD27 signaling *in vitro* following treatment with hexavalent HERA-CD27L, trimeric CD27L or a clinical benchmark anti-human CD27 antibody (1F5) by measuring luciferase activity in a CD27-specific cell-based bioassay (NFκB-luc2/CD27 Jurkat cell bioassay, Promega GmbH). NFκB-luc2/CD27-expessing Jurkat cells were plated in a 96-well plate and incubated at 37°C overnight. The next day, cells were incubated with the indicated concentrations of HERA-CD27L, trimeric CD27L, or anti-CD27 antibody. Productive CD27 signaling induced by treatment with the agonistic compounds drives expression of firefly luciferase in the NFκB-luc2/CD27 Jurkat cells. After 6 h of induction at 37°C, the luciferase assay reagent was added and luminescence (RLU) was measured (Tecan Infinite F500). The fold induction of measured luminescence was calculated by the formula: RLUstimulated/RLUunstimulated control in order to compare multiple experiments.

### Functional binding of hexavalent HERA-CD27L and trimeric CD27L to human, mouse, and cynomolgus monkey CD27-FC

For ELISA assays assessing functional binding of CD27L to its corresponding receptor CD27, coating of microtiter plates was performed with 0.75 μg/mL human or mouse CD27-Fc (Bio-Techne GmbH) or cynomolgus monkey CD27-Fc. Cynomolgus monkey (*Macaca fascicularis*) CD27-Fc fusion protein was generated in house and purified from CHO cells, transfected accordingly, using the two-step protocol described above. After blocking plates with StartingBlock (Life Technologies), wells were incubated with the indicated concentrations of CD27L compounds. CD27L bound to its corresponding receptor was detected via its Strep-Tag II employing Streptactin-HRP (1:5,000, IBA GmbH) and subsequent visualization of the converted Peroxidase-substrate TMB one (Kem-En-Tec Diagnostics) at a wavelength of 450 nm (with a 630 nm correction factor) in an ELISA reader (Thermo Mulitskan Ascent).

### Functional binding of hexavalent HERA-CD27L and trimeric CD27L to primary human T cells

To test functional binding of HERA-CD27L and trimeric CD27L to CD27 expressed by primary cells, peripheral blood mononuclear cells (PBMCs) were isolated from healthy donor buffy coats using standard Ficoll-based density centrifugation protocols. Purified PBMCs were stimulated with soluble anti-CD3 (clone HIT3a, 1 μg/mL) and anti-CD28 (clone CD28.2, 1 μg/mL) antibodies for 3 days. Cells were washed and incubated with 1 μg/mL HERA-CD27L or trimeric CD27L in blocking buffer (PBS, 2% FCS, 1 mg/mL human IgG) for 5 min at 37°C followed by 15 min on ice. Cells were then washed and incubated with rabbit anti-Strep-Tag II (ProScience, Cat. No. 4335), followed by goat F(ab)2 anti-rabbit IgG (biotinylated) (Southern Biotech, Cat. No. 4052-08) and streptavidin-Alexa 488 (Life Technologies, Cat. No. S32354) all in blocking buffer for 20 min on ice prior to staining with anti-human CD27 APC (clone O323) and analysis by flow cytometry.

### *In vitro* T cell activation, proliferation, and differentiation assays

To test the activity of HERA-CD27L and trimeric CD27L on primary human T cells, naïve CD4+ or CD8+ T cells were isolated from PBMCs using indirect magnetic bead-based isolation kits (Cat. No. 130-094-131 and Cat. No. 130-093-244, Miltenyi). Purified T cells were labeled with CFSE (CFSE Cell Division Tracker Kit, BioLegend), resuspended in medium (AIM-V w/o FCS + AlbuMax, Gibco) and stimulated with pre-coated anti-CD3 antibody (overnight, clone OKT3, 1 μg/mL) or medium control. HERA-CD27L or trimeric CD27L, both 100 ng/mL, was added immediately. Between days 2 and 6, T cells were harvested and examined by flow cytometry (analyzed markers as described below). For intracellular staining, cells were treated with PMA (20 ng/ml), Ionomycin (1 μM), and Brefeldin A (1:1,000) at 37°C for 5 h prior to being fixed, permeabilized, stained, and examined by flow cytometry.

### Flow cytometry

For flow cytometry (FCM), cells were labeled with the following antibodies (clone): anti-mouse CD4 (RM4-5), CD8a (53-6.7 or KT15 for tetramer binding studies), and CD44 (IM7) and anti-human CD134 (OX40) (Ber-ACT35), CD137 (4-1BB) (4B4-1), CD25 (BC96), CD27 (O323), CD28 (CD28.2), CD3 (OKT3), CD357 (GITR) (ebioAITR), CD4 (OKT4), CD45RA (HI100), CD45RO (UCHL1), CD8 (SK1), IFN-γ (B27), IL-2 (MQ1-17H12), and TNF-α (MAb11) (all BD Bioscience or Biolegend). Cells were acquired using the FACSCelesta BVR12 (BD Biosciences) or Guava EasyCyte 12 Flow Cytometer (EMD Millipore). Antibody quality was checked and gating was performed using isotype controls. FlowJo Software (10.2) (FlowJo, LLC) was used for the analysis of FCM data.

### Storage, freeze/thaw, heat stress, and pH stability assays

For storage stability, HERA-CD27L was stored at 37 ± 2°C, room temperature or 5 ± 3°C for 1 h, 1 and 4 days, and 2 weeks (at 5 ± 3°C), 1 and 4 days and 2 weeks (at room temperature or 37 ± 2°C) before stability analysis. For freeze/thaw stability, HERA-CD27L was frozen at <-15°C and subsequently thawed at room temperature. Samples were exposed to one, three or five additional freeze/thaw cycles before stability analysis. For pH stability, HERA-CD27L was exposed to pH 2.0, pH 3.0, or pH 4.0 (20 mM Na-citrate/HCl) (Sörensen), pH 7.0 (20 mM phosphate) (Sörensen) or pH 10.0, pH 11.0, pH 12.0 (20 mM glycine/NaOH, 20 mM NaCl) (Sörensen). At 30 min, 2 or 24 h after re-buffering, aliquots were taken and frozen at <-65°C prior to stability analysis. For heat stress, HERA-CD27L was exposed for 10 min in a thermo-block to the following temperatures: 50, 60, 70, 80°C. After exposure to heat and storing these samples at <-15°C, various analytics were performed employing non-heated HERA-CD27L as control.

Procedures used to assess the stability of HERA-CD27L included analytical SEC (HPLC), SDS-PAGE, thermal shift stability assay and determination of binding to the receptor CD27 with an ELISA assay (described above). Analytical SEC of protein samples was performed employing the HPLC device from Agilent (1260 Infinity). Peak heights and peak areas for the main peak as well as for HMWS and LMWS peaks were determined and relative quantities were calculated. For thermal shift stability assays, protein samples were analyzed employing SyPro Orange as a fluorescent dye. In a PCR thermal cycler (MiniOpticon MJ, BioRad), samples were heated from 25 to 95°C with 1°C increments. Fluorescence was monitored at each temperature interval and the melting points of the samples were determined.

### Determination of pharmacokinetic parameters of HERA-CD27L and trimeric CD27L in mice and cynomolgus monkeys

Female CD1 mice or male cynomolgus monkeys were administered with 10 mg/kg body weight (b.w.) or 3 mg/kg b.w., respectively, of HERA-CD27L or trimeric CD27L as a single intravenous (i.v.) injection and whole blood was collected up to 96 h after test item administration. Serum was prepared and HERA-CD27L or trimeric CD27L serum concentrations were quantitated by ELISA assays (assay principle as described above) assessing functional binding of HERA-CD27L or trimeric CD27L to human CD27-Fc. ELISA assays were carried out using reference HERA-CD27L or trimeric CD27L as calibration and control samples. The measured data of the standard concentrations were used to create calibration curves using a five-parameter fit. This enabled the determination of the unknown HERA-CD27L or trimeric CD27L concentrations in the respective serum samples. PK parameters were calculated using the program PK Solutions Version 2.0 for non-compartmental PK data analysis (Summit Research Services). All experimental protocols were approved by the Ethics Committee for Animal Experimentation. The experimental protocols were registered by the regional board in Freiburg, Germany (Regierungspräsidium Freiburg; G-15/41).

### OT-I CD8+ T cell adoptive transfer—analysis of antigen-specific T cell activation *in vivo*

In order to measure antigen-specific T cell responses, we adoptively transferred 2 × 10^6^ splenocytes from T cell receptor (TCR) transgenic “OT-I” donor mice (C57Bl/6 background) i.v. into normal female C57Bl/6 recipient mice as described previously (16). These donor mice have CD8+ T cells that specifically recognize a peptide (SIINFEKL) derived from chicken ovalbumin (OVA) presented by the class I MHC molecule K^b^ (17). Analysis of OT-I CD8+ T cell proliferation was done with whole blood and spleen samples from recipient mice that were injected with OT-I cells on day −1 and challenged with intraperitoneal (i.p.) OVA on day 0 and treated with HERA-CD27L (0.1, 1, and 10 mg/kg b.w.), trimeric CD27L (10 mg/kg b.w.), or vehicle control (PBS) on day 0. For antigen-specific T cell identification on days 6, 9, and 13, we used the iTAg Tetramer H-2 Kb OVA (SIINFEKL) purchased from MBL International together with anti-mouse CD8 (clone KT15, Aviva Systems Biology).

### Analysis of the anti-tumor effect of HERA-CD27L *in vivo* (syngeneic mouse models)

The MC38-CEA anti-tumor efficacy experiment was performed using 6-week-old female C57Bl/6 mice (Strain C57Bl/6NAnNCrl, Charles River). Freshly cultured MC38-CEA tumor cells (1 × 10^6^ in 100 μl PBS) were implanted (day 0) subcutaneously (s.c.) into the left flank of all animals. Following implantation, primary tumor volume was determined twice weekly by caliper measurement using the formula W^2^ × L/2 (L = length and W = the perpendicular width of the tumor, L > W). Eight days after tumor inoculation, mice were randomized into groups of 12 mice per treatment group with a mean primary tumor volume of 65 mm^3^ (range: 25–106 mm^3^). All animals were treated i.v. twice weekly for 2 consecutive weeks (four administrations total on days 8, 12, 15, and 19) starting on the day of randomization. Vehicle control animals were treated with 10 mL/kg b.w. PBS and HERA-CD27L animals were treated at a dose of 1 or 10 mg/kg b.w. Three animals, one in the 1 mg/kg b.w. HERA-CD27L group and two in the 10 mg/kg b.w. HERA-CD27L group, were terminated early due to ethical considerations that were independent of tumor size (observed ulceration). The in-life phase of the study finished 1 day after the last administration of HERA-CD27L (day 20).

The CT26wt anti-tumor efficacy experiment was performed using 6-week-old female BALB/c mice (Strain BALB/cAnNCrl, Charles River). Freshly cultured CT26wt tumor cells (5 × 10^5^ in 100 μl RPMI) were implanted (day 0) s.c. into the right flank of all animals. Eleven days after tumor inoculation, mice were randomized into groups of 12 mice per treatment group with a mean primary tumor volume of 83 mm^3^ (range: 72–100 mm^3^). All animals were treated twice weekly (three administrations total on days 11, 15, and 18) starting on the day of randomization. Vehicle control animals were treated i.v. with 10 mL/kg b.w. PBS, HERA-CD27L animals were treated i.v. at a dose of 1 or 10 mg/kg b.w. and anti-mouse PD1 (CD279, clone RMP1-14, BioXcell) animals were treated i.p. at a dose of 10 mg/kg b.w. One animal from the control group was terminated on day 22 due to tumor size. One to four animals in each group were terminated early due to ethical considerations that were independent of tumor size (observed ulceration). The in-life phase of the study finished on day 25 following tumor implantation.

At the end of the studies, tumor samples and spleens were harvested and tumor weights were measured. Experimental protocols were approved by the Ethics Committee for Animal Experimentation. The experimental protocol was registered by the regional board in Freiburg, Germany (Regierungspräsidium Freiburg; G-15/41).

### Tissue isolation and sample preparation from *in vivo* assays

For tissue isolation, spleens, tumors, and lymph nodes (LNs; axillary, brachial, and inguinal) were removed and organs were processed with a gentleMACS Dissociator (Miltenyi) and filtered to prepare single-cell suspensions. Whole venous blood was collected in lithium-heparin coated capillary tubes. Ammonium chloride–potassium bicarbonate lysis buffer was used to lyse erythrocytes in spleen and blood samples.

### Determination of equilibrium binding constants (K_D_)

The equilibrium binding constants (K_D_) of HERA-CD27L and trimeric CD27L with human and mouse CD27-Fc were calculated based on kinetic binding data (k_on_ and k_off_) determined with an automated biosensor system (Attana A100). The A100 allows to investigate molecular interactions in real-time based on the Quartz Crystal Microbalance (QCM) technique. For this purpose, the respective recombinant human and mouse CD27-Fc were immobilized on the surface of a carboxyl-activated QCM-chip. HERA-CD27L and trimeric CD27L were used as soluble analytes at different concentrations. Binding and dissociation was analyzed in real time, and the respective K_D_ was calculated.

### Statistics

Statistical analysis of experimental data was performed using Prism software (GraphPad). Employed statistical tests and corresponding parameters are mentioned in the Figure legends. In general, one-way (or two-way for tumor growth studies) ANOVA tests were performed followed by *post-hoc* Bonferroni multiple comparisons analysis. Results were considered statistically significant if *p* < 0.05.

## Results

### Structure, design, and production of a novel *he*xavalent TNFRSF receptor *a*gonist of CD27 (HERA-CD27L)

**Figure 1:**
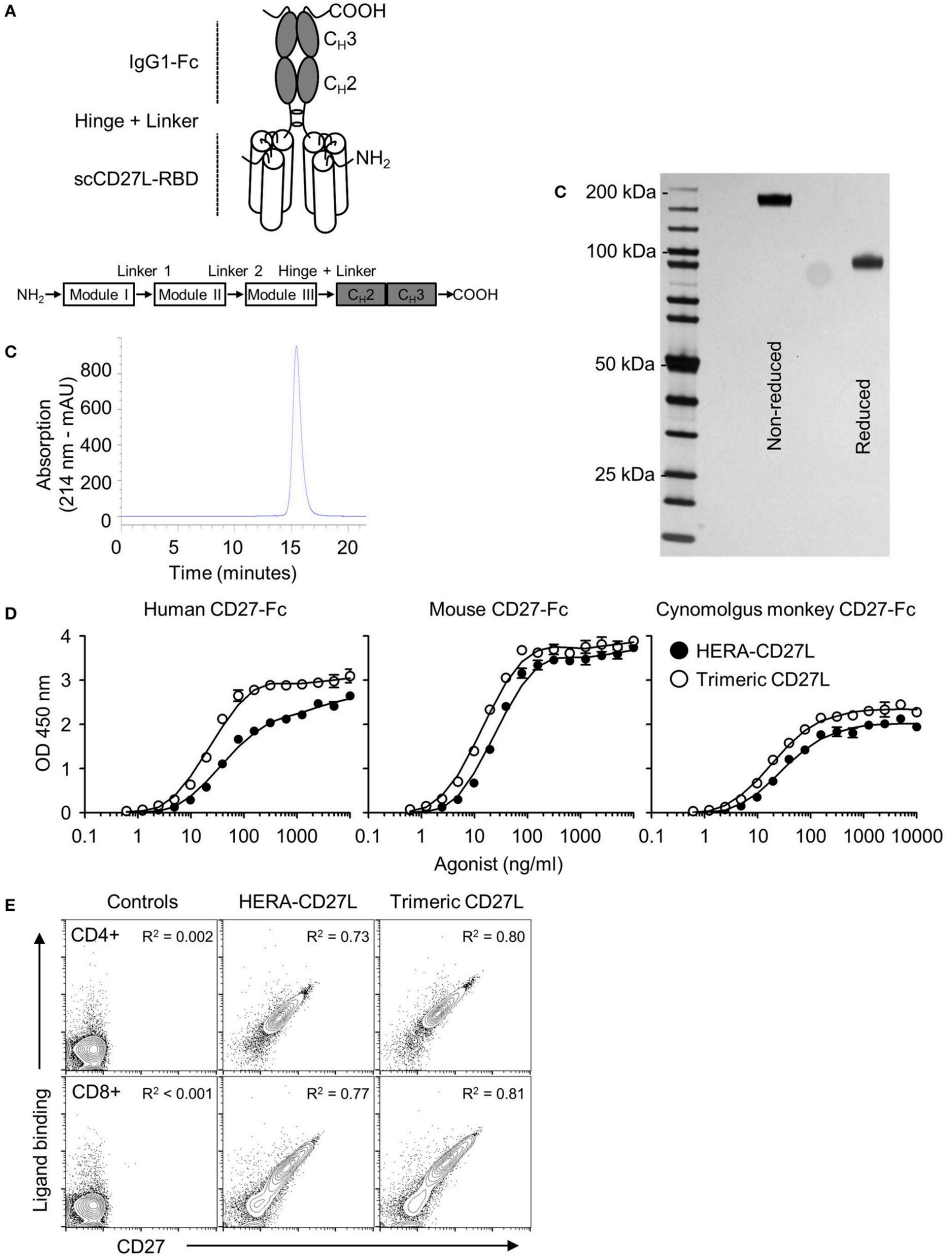
Structure, design, and production of HERA-CD27L. **(A)** Schematic depiction of the structure of the hexavalent HERA-CD27L. Three copies of a CD27L (CD70) protomer sub-sequence (called the CD27L-receptor binding domain or CD27L-RBD) were combined into one polypeptide chain. This generated a trivalent scCD27L-RBD which was fused to the Fc part of a human IgG1-mutein to create a hexavalent scCD27L-RBD dimer. **(B)** Purification was accomplished by a two-step process combining AFC followed by preparative SEC. For analytical SEC, purified HERA-CD27L was detected by online measurement of absorption at 214 nm. Content of monomer and aggregates was calculated as the AUC from the elution profile of the SEC. HERA-CD27L eluted as a single peak and showed no detectable aggregates. **(C)** Purity and aggregation status of purified hexavalent HERA-CD27L was also assessed by non-reducing and reducing SDS-PAGE. **(D)** ELISA showing binding of hexavalent HERA-CD27L and trimeric CD27L to immobilized human, mouse and cynomolgus monkey CD27-Fc. Plate-bound CD27-Fc was probed with the indicated formats and concentrations. Receptor-bound HERA-CD27L and CD27L was detected via a Strep-Tag II-specific antibody conjugated to horseradish peroxidase. Values are mean OD (*n* = 3) ± S.D. at a wavelength of 450 nm (with a 630 nm correction) with a best-fit line added for clarity. Representative data from at least three independent experiments are shown. **(E)** Binding of hexavalent HERA-CD27L and trimeric CD27L to purified human CD4+ and CD8+ T cells, top row and bottom row, respectively. HERA-CD27L and trimeric CD27L (both 1 μg/mL) were incubated with purified human CD4+ or CD8+ T cells followed by an anti-Strep-Tag II antibody to visualize ligand binding and an anti-CD27 antibody (clone O323) to visualize receptor expression by flow cytometry. “Control” samples were incubated with anti-Strep-Tag II antibody only (no ligand) plus isotype antibody. Representative plots, gated on live single cells, from at least three independent experiments are shown. Pearson correlation coefficients were determined for each group (all groups were *P* < 0.0001) and the *R*^2^ values are shown.

In order to investigate the biological activities of different CD27 agonist formats, we expressed and purified a hexavalent single chain CD27L-Fc (designated HERA-CD27L) as well as a trimeric CD27L in CHO-S cells. The construction principles are outlined in Figure 1A and Supplementary Figure S1A. All preclinical HERA ligands included a C-terminal Strep-Tag II for purification of the proteins. As shown by SDS-PAGE and analytical SEC, the lab scale production process yielded defined and stable products devoid of contaminating proteins and product related aggregates (Figures 1B,C; Supplementary Figures S1B,C). In addition, the activity and stability of HERA-CD27L, measured by receptor binding and a thermal shift assay, was tested under a variety of different temperature and pH conditions and it was found to be very stable and suitable for standard large-scale production processes (Supplementary Figure S1D and Supplementary Table S1).

### Binding of HERA-CD27L and trimeric CD27L to human, mouse, and cynomolgus monkey CD27

Purified HERA-CD27L and trimeric CD27L were tested for binding to recombinant human, mouse, and cynomolgus monkey CD27 by ELISA. As shown in Figure 1D, both molecules were able to bind to immobilized human and mouse as well as cynomolgus monkey CD27-Fc demonstrating functional assembly of the respective receptor binding domains. A summary of binding constants for HERA-CD27L and trimeric CD27L binding to human and mouse CD27-Fc is shown in Supplementary Table S2. These data confirm that both mouse and cynomolgus monkey are relevant species for further studies.

To test functional binding to primary T cells and to test the compatibility of ligand binding and anti-CD27 antibody binding, HERA-CD27L and trimeric CD27L were incubated with purified human CD4+ or CD8+ T cells followed by an anti-Strep-Tag II antibody to visualize ligand binding and an anti-CD27 antibody (clone O323) to visualize receptor expression. As shown in Figure 1E, there is a direct correlation between ligand binding and receptor expression on both CD4+ and CD8+ T cells. In addition, we found that ligand binding and anti-receptor antibody binding, at least for clone O323, were fully compatible since reversing the incubation order, antibody followed by either ligand, resulted in the same expression patterns (data not shown).

### HERA-CD27L enhances human T cell activation following stimulation *in vitro*

**Figure 2:**
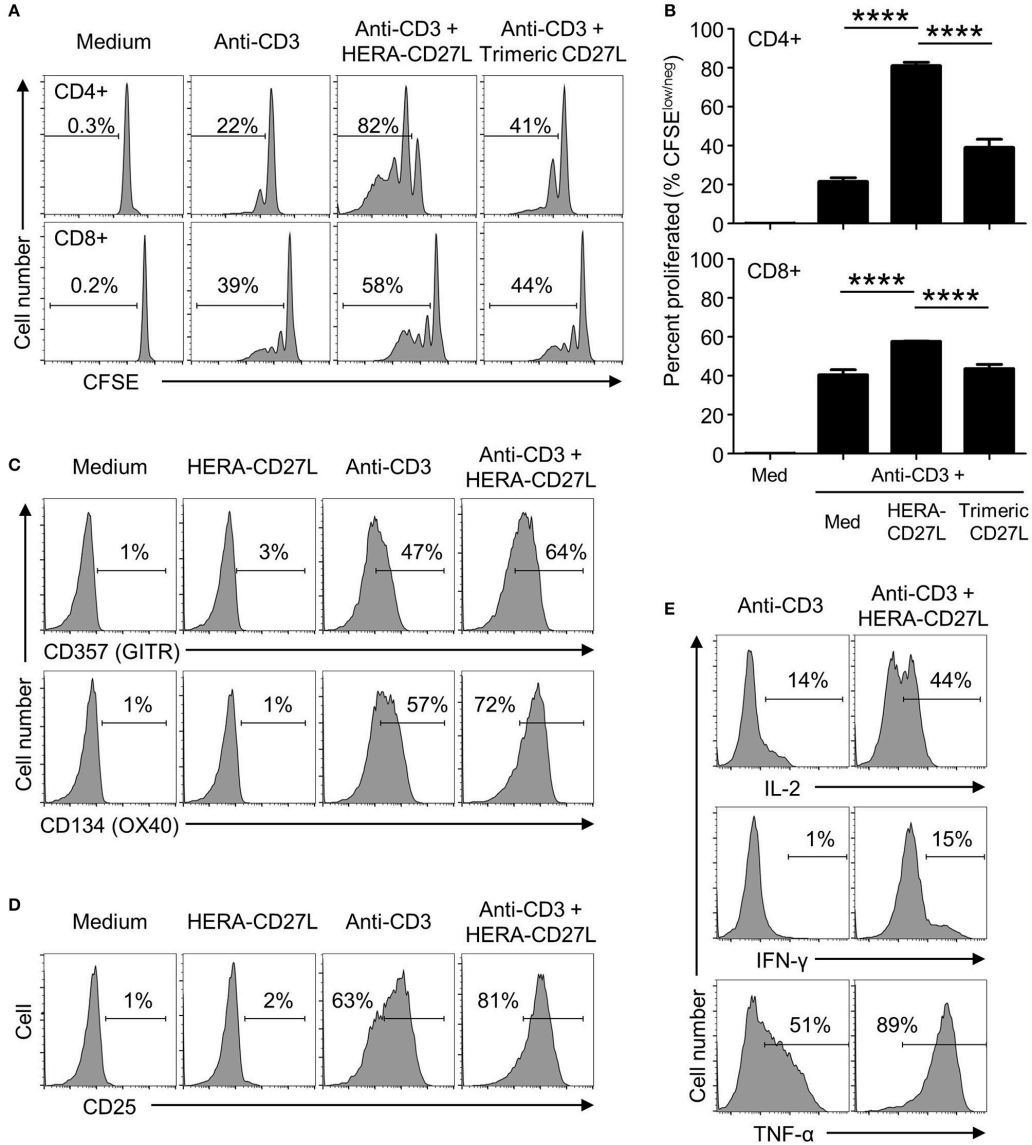
HERA-CD27L enhances human T cell activation following stimulation *in vitro*. **(A,B)** Naïve CD4+ or CD8+ T cells were isolated from the peripheral blood of healthy volunteers, labeled with CFSE and stimulated with medium control or anti-CD3 antibody in the presence of HERA-CD27L, trimeric CD27L (both 100 ng/mL), or vehicle control (PBS), as indicated. On day 5 (CD4+) or day 4 (CD8+), T cells were harvested and examined by flow cytometry. Representative histograms **(A)** and quantified data **(B)** are shown. The *p*-values represent comparisons between samples using a one-way ANOVA plus *post-hoc* Bonferroni multiple comparisons test. ****p < 0.0001. Although not labeled, all groups were significantly different from the medium alone group. **(C,D)** Naïve CD4+ T cells were stimulated with anti-CD3 antibody or medium control in the presence/absence of HERA-CD27L (100 ng/mL), as indicated. On day 5, T cells were harvested, stained for surface expression of **(C)** CD357 (GITR) and CD134 (OX40) or **(D)** CD25 (IL-2Rα) and examined by flow cytometry. **(E)** Naïve CD4+ T cells were stimulated with anti-CD3 antibody in the presence/absence of HERA-CD27L (100 ng/mL), as indicated. On day 6, T cells were harvested, fixed, permeabilized, and stained for intracellular expression of IL-2, IFN-γ, and TNF-α and examined by flow cytometry. Numbers indicate the percentage of cells within the defined region. Representative histograms, gated on live single cells, from the median sample of triplicates from at least three independent experiments are shown.

To examine the biological activity of HERA-CD27L and trimeric CD27L, we isolated naïve T cells from the peripheral blood of healthy volunteers and stimulated them in the presence of our CD27 ligands. Naïve T cells require a specific stimulatory signal transmitted through the TCR as well as a second set of “co-stimulatory” signals in order to achieve increased proliferation and differentiation. Naïve CD4+ and CD8+ T cells were stimulated with plate-bound anti-CD3 antibody (1 μg/mL) (“signal one”) in the presence of HERA-CD27L or trimeric CD27L (both 100 ng/mL). Addition of HERA-CD27L increased the proliferative response of both CD4+ and CD8+ T cells stimulated by anti-CD3 antibody (Figures 2A,B). In contrast, the trimeric CD27L showed a much weaker effect on T cell proliferation, especially the CD8+ T cell population.

Corresponding changes in the expression of the differentiation markers, CD45RA and CD45RO, were also observed following treatment with HERA-CD27L in combination with anti-CD3 antibody (Supplementary Figure S2A). In addition to these classic differentiation markers, CD4+ T cells activated in the presence of HERA-CD27L also upregulated surface expression of CD357 (GITR) and CD134 (OX40) (Figure 2C) while CD8+ T cells increased expression of CD137 (4-1BB) (Supplementary Figure S2B), three other members of the TNFRSF. In order to understand the mechanism of improved proliferation following treatment with HERA-CD27L, we investigated the IL-2 pathway. As shown in Figures 2D,E, both surface expression of CD25 (IL-2Rα) and intracellular accumulation of IL-2 increased following HERA-CD27L treatment. There was also an increase in intracellular accumulation of the inflammatory cytokines IFN-γ and TNF-α (Figure 2E).

Importantly, treatment with HERA-CD27L alone did not produce any response in unstimulated T cells, suggesting that an antigen-specific and TCR-mediated signal is required for the CD27-mediated enhancement of T cell activity (Figures 2C,D; Supplementary Figure S2A).

### CD27 expression kinetics following treatment with HERA-CD27L *in vitro*

**Figure 3:**
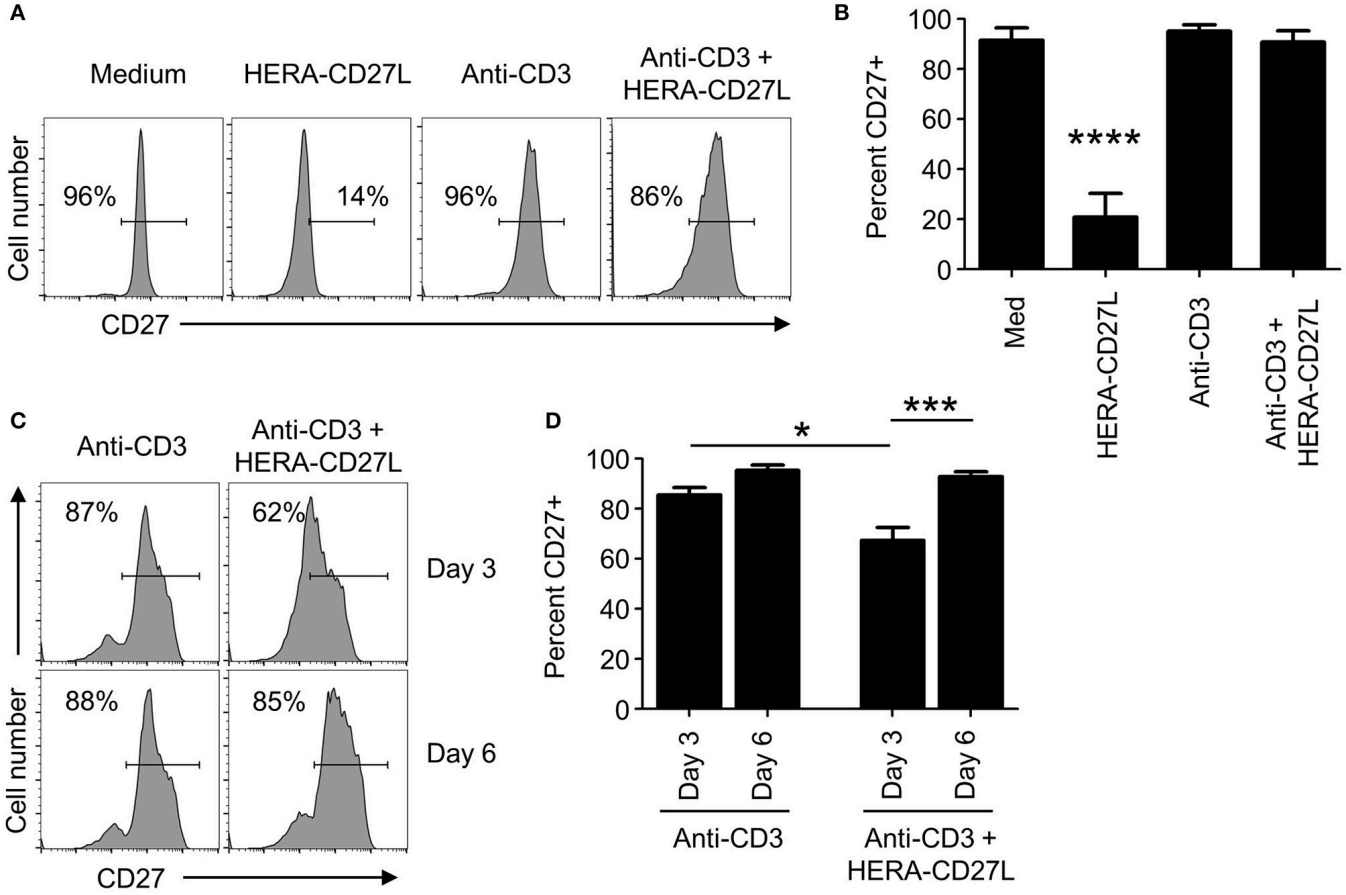
CD27 expression kinetics following treatment with HERA-CD27L *in vitro*. **(A,B)** Naïve CD4+ T cells were isolated from the peripheral blood of healthy volunteers and stimulated with anti-CD3 antibody or medium control in the presence/absence of HERA-CD27L (100 ng/mL), as indicated. On day 5, T cells were harvested, stained for surface expression of CD27 and examined by flow cytometry. Representative histograms **(A)** and quantified data **(B)** are shown. The *p*-value represents comparisons between the HERA-CD27L alone group and all other samples using a one-way ANOVA plus *post-hoc* Bonferroni multiple comparisons test. ****p < 0.0001. **(C,D)** Naïve CD4+ T cells were stimulated with anti-CD3 antibody in the presence/absence of HERA-CD27L (100 ng/mL), as indicated. On days 3 and 6 T cells were harvested, stained for surface expression of CD27 and examined by flow cytometry. Representative histograms **(C)** and quantified data **(D)** are shown. The *p*-values represent comparisons between samples using a one-way ANOVA plus *post-hoc* Bonferroni multiple comparisons test. *p < 0.05, ***p < 0.001. Numbers indicate the percentage of cells within the defined region. Representative histograms, gated on live single cells, from the median sample of triplicates from at least three independent experiments are shown.

Although no proliferative/differentiative responses were visible following HERA-CD27L treatment of unstimulated naïve CD4+ T cells *in vitro*, an almost complete loss of surface CD27 expression was observed on day 5 after treatment (Figures 3A,B). In contrast, stimulated T cells treated with HERA-CD27L showed similarly high levels of CD27 on their cell surface as control T cells. In order to better understand the kinetics of CD27 expression following T cell activation and treatment with HERA-CD27L, we examined earlier time points following activation. As seen in Figures 3C,D, activated T cells treated with HERA-CD27L had reduced expression of CD27 on day 3 following stimulation but expression was similar to anti-CD3 treatment by day 6. These data suggest that the exposure to the CD27 agonist, relative to the TCR signal, could play a critical role in maintaining CD27 expression and, consequently, sensitivity to CD27-based therapy. Therefore, we decided to investigate the PK characteristics of HERA-CD27L and trimeric CD27L *in vivo*.

### Pharmacokinetics of HERA-CD27L and trimeric CD27L in mouse and cynomolgus monkey

Since human HERA-CD27L and trimeric CD27L also bind to mouse and cynomolgus monkey CD27, we conducted PK studies in both of these relevant species. Female CD-1 mice were treated with HERA-CD27L or trimeric CD27L (both 10 mg/kg b.w.) as a single i.v. bolus injection. The terminal serum half-life of HERA-CD27L was calculated to be 14.5 h, while the trimeric CD27L had a half-life of 8.5 h (Supplementary Figure S3A and Supplementary Table S3). A pilot study performed in cynomolgus monkeys showed that HERA-CD27L (3 mg/kg b.w.) had a similar half-life as seen in mice, approximately 11.4 h (Supplementary Figure S3B and Supplementary Table S3). Importantly, no toxicological signs related to the administration of HERA-CD27L were observed during the in-life phase of the study (data not shown).

### HERA-CD27L significantly boosts the antigen-specific CD8+ T cell response *in vivo*

**Figure 4:**
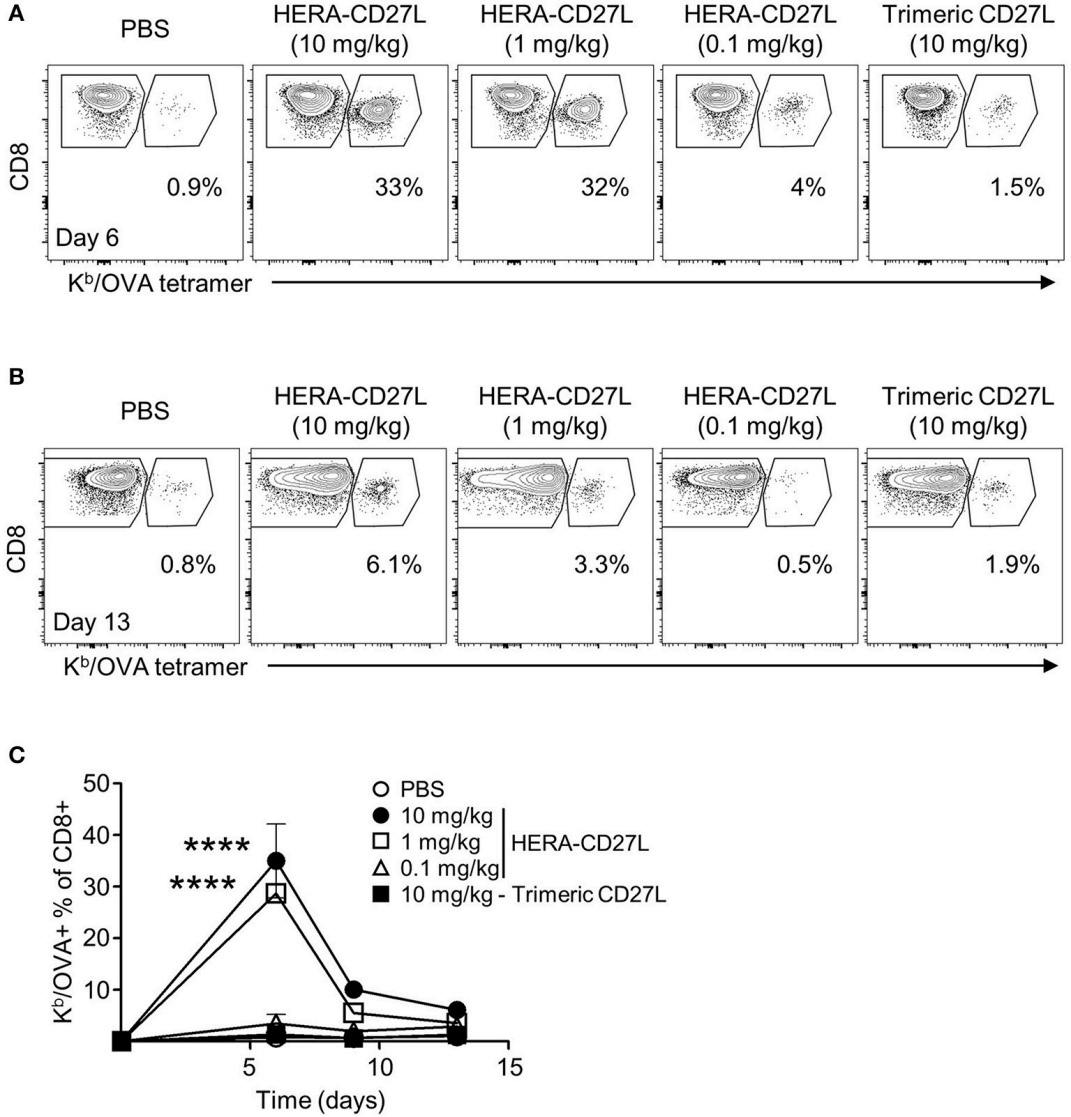
HERA-CD27L significantly boosts the antigen-specific CD8+ T cell response *in vivo*. **(A–C)** Female C57Bl/6 recipient mice were adoptively transferred with 2 × 10^6^ CD8+ OT-I T cells, challenged with OVA protein and treated with a single injection of HERA-CD27L (0.1, 1, and 10 mg/kg b.w.), trimeric CD27L (10 mg/kg b.w.) or vehicle control (PBS). Serial blood samples were obtained from each animal over a 2-week period and OT-I T cells were quantified by flow cytometry using CD8 and a K^b^/OVA tetramer. Representative time points are shown **(A)** for day 6 and **(B)** for day 13 and **(C)** the entire time course is quantified. **(C)** Day 0 value represents historical data following adoptive transfer of 2 × 10^6^ CD8+ OT-I T cells. Each symbol represents the mean (*n* = 3) ± S.D. Gated regions indicate CD8+ OT-I T cells (right polygon) and endogenous/recipient CD8+ T cells (left polygon). Numbers indicate the percentage of cells within the OT-I T cell region (CD8+ K^b^/OVA+). Representative contour plots, gated on live single CD8+ T cells, from the median sample of triplicates from at least four independent experiments are shown. A two-way ANOVA plus *post-hoc* Bonferroni multiple comparisons analysis was conducted to compare the effects of treatment and time on antigen-specific T cell clonal expansion. The *p*-values displayed represent comparisons between 1 and 10 mg/kg HERA-CD27L and PBS. ****p < 0.0001.

In Figure 2, we showed that HERA-CD27L enhanced T cell activation *in vitro*. In order to test the activity of HERA-CD27L and trimeric CD27L in a more physiologically relevant system, we used a variation of the classic antigen-specific adoptive transfer system (16). This system allows for the investigation of a more realistically sized antigen-specific T cell population (around 1 percent) while simultaneously having a large population of non-specific T cells to serve as control T cells. Since most T cells express CD27, there is potential for non-specific and bystander T cell activation. These dangers were examined by comparing OVA-specific and non-specific T cells in the same environment using an OVA-specific MHC/peptide tetramer. Since the OVA-tetramer positive T cell frequency in wildtype C57Bl/6 mice is very low [on average, < 1,000 cells per mouse (18)], we adoptively transferred OVA-specific CD8+ “OT-I” T cells to increase the resolution of the tetramer staining. Wildtype C57Bl/6 mice were adoptively transferred with 2 × 10^6^ OVA-specific CD8+ “OT-I” T cells and challenged with OVA protein plus a single injection of HERA-CD27L (0.1, 1, or 10 mg/kg b.w.) or trimeric CD27L (10 mg/kg b.w.). Serial blood samples were obtained following challenge and representative examples of OT-I T cell clonal expansion are shown in Figure 4A (day 6) and Figure 4B (day 13). As shown in Figure 4C, antigen-specific T cells underwent significant clonal expansion during the first week following antigen challenge and treatment with the higher two doses of HERA-CD27L. In contrast, treatment with 0.1 mg/kg b.w. HERA-CD27L or 10 mg/kg b.w. trimeric CD27L resulted in a minimal T cell response. Along with increased clonal expansion, OT-I T cells also upregulated surface expression of CD44, a differentiation/maturation marker, following treatment with HERA-CD27L (Supplementary Figure S4A). Importantly, the endogenous CD4+ and CD8+ T cells did not respond to treatment with HERA-CD27L (Supplementary Figure S4B).

### HERA-CD27L demonstrates significant *in vivo* efficacy in two syngeneic mouse tumor models

Although T cell activation assays are good for proof of concept and mechanism of actions studies, it is critically important for anti-cancer compounds to show *in vivo* efficacy in mouse tumor models. Two commonly used colon carcinoma models, MC38-CEA (C57Bl/6) and CT26wt (BALB/c), were chosen to investigate the potency of HERA-CD27L treatment. These two models have been shown to be highly immunogenic and generally responsive to immunotherapy. In addition, the two distinct genetic backgrounds gave an opportunity to address tumor efficacy in the context of their propensities toward different immune polarization, for example, type 1 versus type 2 immunity, since CD27-CD70 interactions have been often associated with type 1 responses (19).

**Figure 5:**
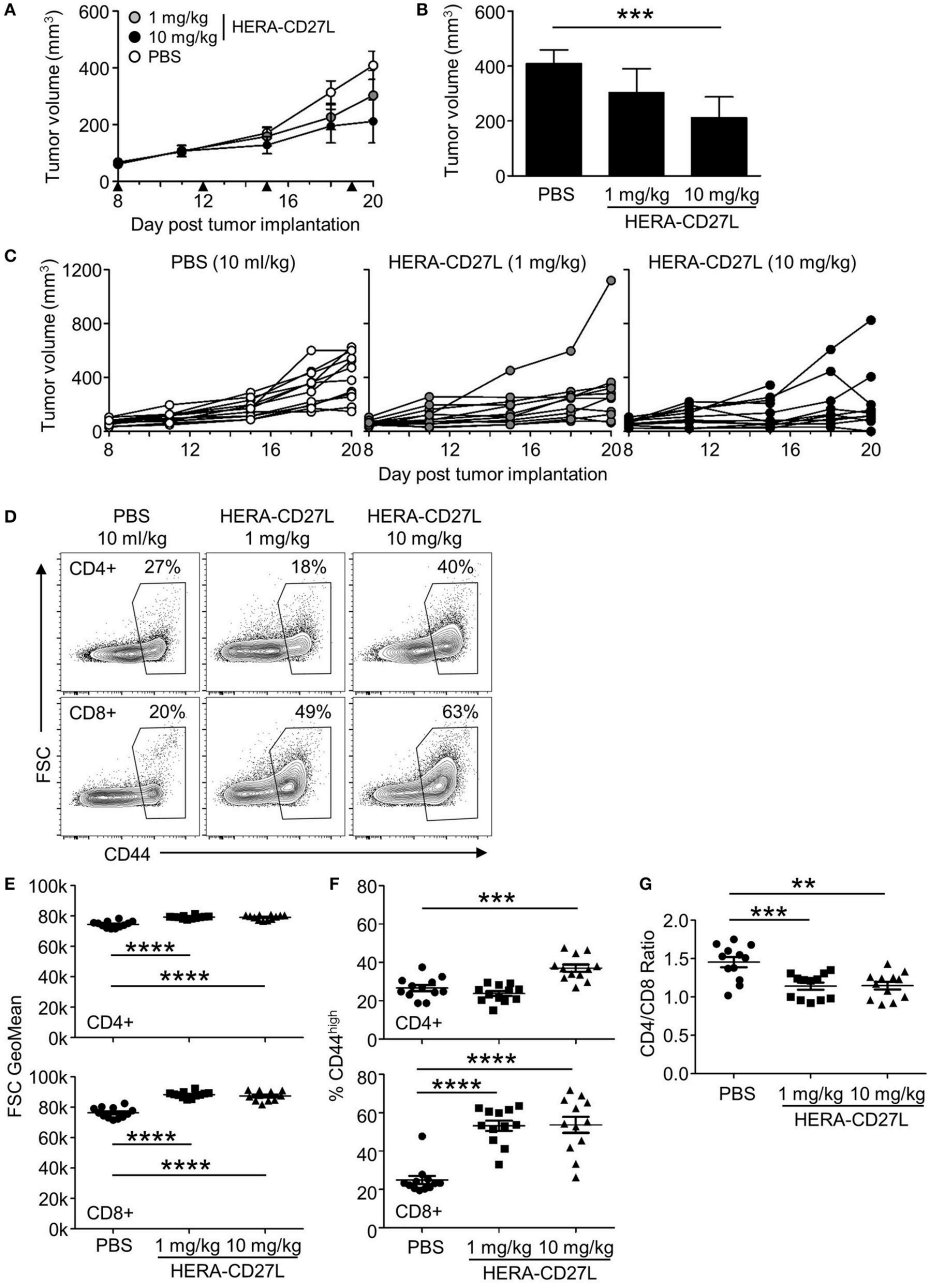
HERA-CD27L demonstrates significant *in vivo* efficacy in mice as a single agent in the MC38-CEA tumor model. **(A–G)** Freshly cultured MC38-CEA tumor cells (1 × 10^6^ in 100 μl PBS) were implanted s.c. into the left flank of 6-week-old female C57Bl/6 mice. Tumor volume was determined twice weekly by caliper measurement. Mice were randomized on day 8 into groups of 12 mice per treatment group with a mean primary tumor volume of 65 mm^3^. All animals were treated i.v. with 1 or 10 mg/kg b.w. of HERA-CD27L or vehicle control (PBS) on days 8, 12, 15, and 19, indicated by the black triangles. The in-life phase of the study finished 1 day after the last administration of HERA-CD27L (day 20). Three animals, 1 in the 1 mg/kg b.w. HERA-CD27L group and 2 in the 10 mg/kg b.w. HERA-CD27L group, were terminated early due to ethical considerations that were independent of tumor size. **(A,B)** Each symbol or column represents the mean (*n* = 12) ± S.D. **(B)** Shows the tumor volume on day 20. **(C)** Tumor size for all individual animals is shown. A two-way ANOVA plus *post-hoc* Bonferroni multiple comparisons analysis was conducted to compare the effects of treatment and time on tumor growth. ***p < 0.001. **(D–G)** On day 20, spleens were harvested, stained for surface marker expression and examined by flow cytometry. **(D)** Representative contour plots, gated on live single CD4+ or CD8+ T cells, from the median sample of *n* = 12 from at least two independent experiments are shown. Numbers indicate the percentage of cells within the defined region. **(E–G)** Horizontal lines indicate the mean (*n* = 12) ± S.D for each group. The *p*-values represent comparisons between samples using a one-way ANOVA plus *post-hoc* Bonferroni multiple comparisons test. **p < 0.01, ***p < 0.001, ****p < 0.0001. **(E)** The forward scatter (FSC) geometric mean is shown for CD4+ or CD8+ T cells, top and bottom rows, respectively. **(F)** The percentage of CD44high cells is shown for CD4+ or CD8+ T cells, top and bottom rows, respectively. **(G)** The ratio of CD4+ to CD8+ T cells is shown.

MC38-CEA tumor cells were implanted s.c. and animals were randomized into groups 8 days later. All animals were treated i.v. with 1 or 10 mg/kg b.w. of HERA-CD27L or vehicle control (PBS) on days 8, 12, 15, and 19. As shown in Figures 5A–C, HERA-CD27L treatment produced dose-dependent tumor-growth inhibition (TGI) compared to the control group, including 48% TGI at 10 mg/kg b.w. HERA-CD27L (*p* < 0.05). In addition, both CD4+ and CD8+ T cells isolated from the spleens of tumor-bearing mice on day 20 showed evidence of activation and differentiation (Figures 5D–F). CD4+ T cells were significantly larger at both doses tested, while treatment with 10 mg/kg b.w. HERA-CD27L resulted in an increased proportion of cells expressing high levels of CD44. The response in the CD8+ T cell population was more pronounced and they showed significant blasting and differentiation at both doses tested. In addition to these phenotypic changes, there was a significant increase in the proportion of CD8+ T cells relative to CD4+ T cells, B cells, and all hematopoietic cells (Figure 5G and data not shown).

**Figure 6:**
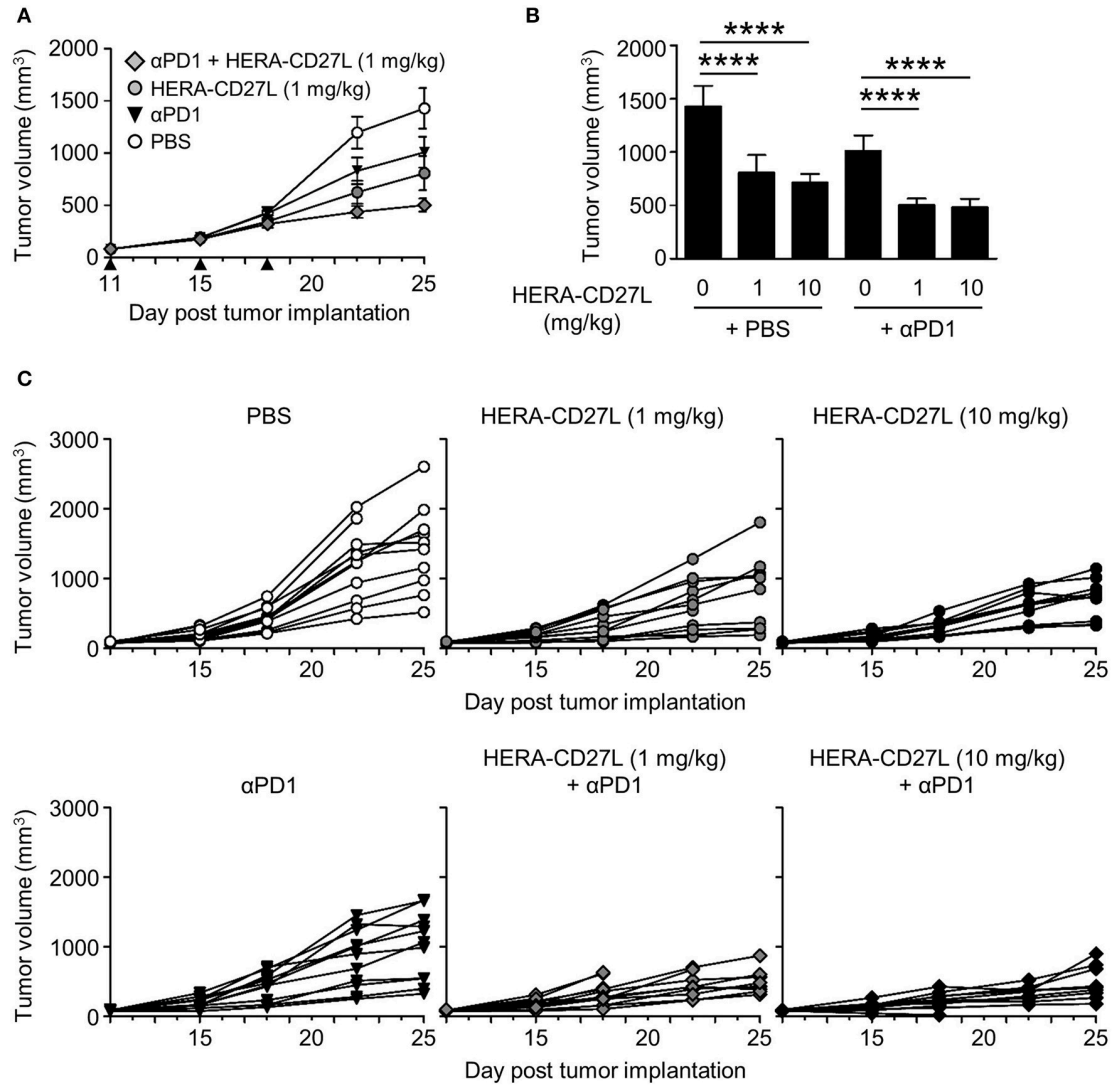
HERA-CD27L demonstrates significant *in vivo* efficacy as a single agent as well as in combination with PD-1 blockade in the syngeneic CT26wt mouse tumor model. **(A–C)** Freshly cultured CT26wt tumor cells (5 × 10^5^ in 100 μl RPMI) were implanted s.c. into the right flank of 6-week-old female BALB/c mice. Tumor volume was determined twice weekly by caliper measurement. Mice were randomized on day 11 into groups of 12 mice per treatment group with a mean primary tumor volume of 83 mm^3^. All animals were treated with 1 or 10 mg/kg b.w. of HERA-CD27L (i.v.), 10 mg/kg b.w. anti-PD-1 antibody (i.p.), a combination of 1 or 10 mg/kg b.w. of HERA-CD27L (i.v.), and 10 mg/kg b.w. anti-PD-1 antibody (i.p. application 2 h after HERA-CD27L application) or vehicle control (PBS) (i.v.) on days 11, 15, and 18, indicated by the black triangles. One to four animals in each group were terminated early due to ethical considerations that were independent of tumor size. The in-life phase of the study finished on day 25 following tumor implantation. **(A,B)** Each symbol or column represents the mean (*n* = 12) ± S.D. Only the low dose (1 mg/kg) HERA-CD27L groups are shown in **(A). (C)** Tumor size for all individual animals is shown. A two-way ANOVA plus *post-hoc* Bonferroni multiple comparisons analysis was conducted to compare the effects of treatment and time on tumor growth. ****p < 0.0001. The PBS alone group is significantly different from all other groups at the final two time points (days 22 and 25). **(B)** Shows tumor volume on day 25, labeled *p*-values are consistent with day 22 as well.

CT26wt tumor cells were implanted s.c. and animals were randomized into groups 11 days later. All animals were treated with 1 or 10 mg/kg b.w. of HERA-CD27L (i.v.), 10 mg/kg b.w. anti-PD-1 antibody (i.p.) or vehicle control (PBS) (i.v.) on days 11, 15, and 18. In this model, 1 mg/kg b.w. of HERA-CD27L alone showed 43% TGI and anti-PD-1 alone showed 29% TGI (Figures 6A–C). In addition, HERA-CD27L plus anti-PD-1 combination therapy, at the low dose, increased TGI to 66%. Interestingly, in this model, there was no evidence of an increase in peripheral T cell activation at the end of the study (day 25, Supplementary Figures S5A–D).

### HERA-CD27L demonstrates significantly enhanced activity compared to a clinical benchmark agonistic anti-CD27 antibody

Due to the role that TNFRSF receptors play in initiating and prolonging anti-tumor immunity, multiple approaches have been developed to create TNFRSF receptor agonists. The most common approach has been to generate anti-receptor antibodies. Although antibodies have been shown to be effective inhibitors of signaling pathways, they have mixed results as agonists due to their limited binding domains and mixed mechanism of action.

**Figure 7:**
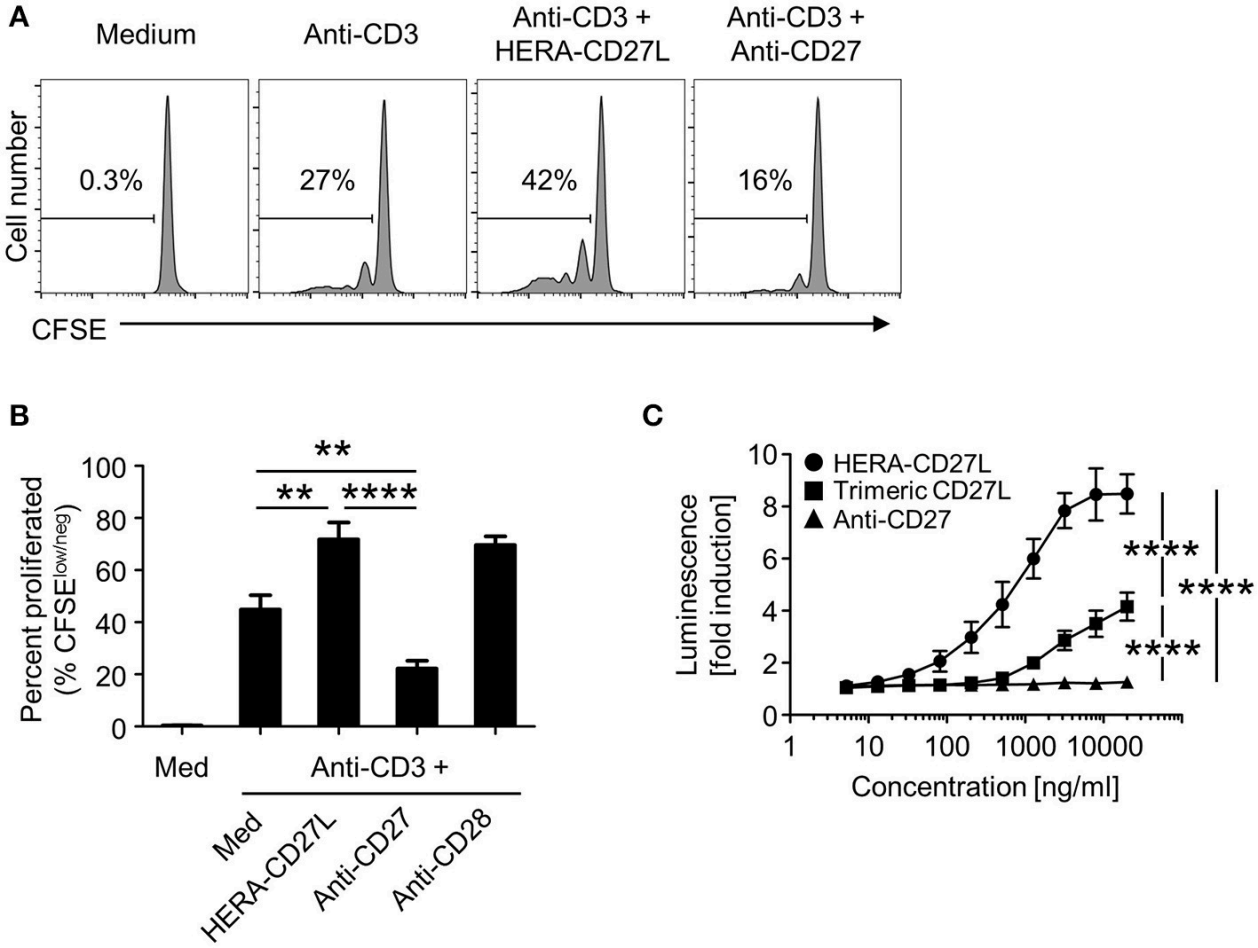
HERA-CD27L demonstrates significantly enhanced activity compared to a clinical benchmark anti-CD27 antibody. **(A,B)** Naïve CD4+ were isolated from the peripheral blood of healthy volunteers, labeled with CFSE and stimulated with anti-CD3 antibody or medium control in the presence of HERA-CD27L (100 ng/mL) or anti-CD27 antibody (1 μg/mL), as indicated. On day 5, T cells were harvested and examined by flow cytometry. Representative histograms **(A)**, gated on live single cells, from the median sample (*n* = 3) from a representative experiment are shown. Numbers indicate the percentage of cells within the defined region (i.e., proliferating cells). **(B)** Quantification of data from at least three independent experiments. The *p*-values represent comparisons between samples using a one-way ANOVA plus *post-hoc* Bonferroni multiple comparisons test. **p < 0.01, ****p < 0.0001. Although not labeled, all groups were significantly different from the medium alone group. The anti-CD3/CD28 group was not included in the statistical analysis because it was only from 1 experiment (*n* = 3). However, the data are consistent with our historical results. **(C)** NFκB-luc2/CD27-expressing Jurkat cells were incubated with the indicated concentrations of HERA-CD27L, trimeric CD27L, or anti-CD27 antibody at 37°C. After 6 h, luminescence was measured and the fold induction in luciferase activity was calculated in order to compare multiple experiments. Data are shown as mean values (±SEM) from three independent experiments. A two-way ANOVA plus *post-hoc* Bonferroni multiple comparisons analysis was conducted to compare the effects of treatment and concentration on luminescence. The *p*-values displayed represent comparisons between groups at the highest concentration. ****p < 0.0001. Although not labeled, HERA-CD27L is significantly different from the other two groups at all concentrations above 100 ng/ml. The trimeric CD27L is significantly different from the anti-CD27 antibody at the highest two concentrations.

As we have shown in Figures 2, 4, the hexavalent HERA-CD27L was more effective than the trimeric CD27L. To test the hypothesis that bivalent compounds, like antibodies, would have even more trouble clustering a sufficient number of receptors to transmit a productive signal, we produced a clinical benchmark anti-human CD27 antibody to compare *in vitro*. In order to compare the hexavalent HERA-CD27L to the bivalent antibody, naïve CD4+ T cells were stimulated with plate-bound anti-CD3 antibody (1 μg/mL) in the presence of HERA-CD27L (100 ng/mL) or anti-CD27 antibody (1 or 5 μg/mL). Addition of HERA-CD27L significantly increased the proliferative response of T cells stimulated by anti-CD3 antibody (Figures 2A,B, 7A,B). In contrast, treatment with the anti-CD27 antibody resulted in a significantly weaker proliferative response, even compared to anti-CD3 antibody alone (Figures 7A,B, 1 μg/mL; data not shown, 5 μg/mL). In order to understand how treatment with a supposedly agonistic clinical benchmark antibody could result in decreased T cell proliferation, we tested productive CD27 signaling using a reporter cell assay. NFκB-luc2/CD27-expressing Jurkat cells were incubated with various concentrations of HERA-CD27L, trimeric CD27L, or anti-CD27 antibody and after 6 h of culture, luminescence was measured. As shown in Figure 7C, treatment with HERA-CD27L and trimeric CD27L resulted in high and intermediate, respectively, reporter activity that was consistent with the proliferation results. Interestingly, the anti-CD27 antibody failed to show any CD27 signaling activity across a wide range of concentrations.

## Discussion

Immunotherapeutic strategies targeting stimulatory TNFRSF receptors to promote anti-tumor immune responses have tremendous potential and CD27 is an important candidate due to its unique role in both initiating as well as maintaining T cell responses. We have shown here that HERA-CD27L significantly boosts antigen-specific T cell activity while having no effect on non-specific T cells. This point is critical since the majority of T cells express CD27 and could, therefore, respond to HERA-CD27L treatment. This also makes HERA-CD27L particularly suitable for combination therapy with strategies that provide additional antigen-specific immune cells, such as vaccination and adoptive cellular transfer (10, 20, 21).

Here we describe the design and production of a novel hexavalent agonist of CD27. The HERA-CD27L production process resulted in a defined and stable product devoid of contaminating impurities and product-related aggregates. Furthermore, HERA-CD27L was tested under a variety of different conditions and found to be stable and suitable for standard large-scale production processes. Binding studies confirmed previously published reports that the fully human HERA-CD27L binds to both human and mouse CD27 (5). Our data further showed that HERA-CD27L can also bind to cynomolgus monkey CD27 which indicates that both mouse and cynomolgus monkey can be used as relevant species for further studies. To further explore this topic, small scale PK studies were performed in both species. These studies showed that HERA-CD27L had a similar terminal serum half-life in cynomolgus monkeys and mice, ~11–15 h. Critically, no toxicological signs related to the administration of HERA-CD27L were observed during the in-life phase of these studies.

In order to understand the biological activity of HERA-CD27L, naïve human T cells were stimulated *in vitro* in the presence of HERA-CD27L or the trimeric CD27L. In all assays performed, treatment with the hexavalent HERA-CD27L significantly boosted activation, proliferation and differentiation of stimulated T cells. Furthermore, the hexavalent molecule was always superior to the trimeric CD27L. This response can be partially explained by the concurrent increase in IL-2 and CD25 expression by HERA-CD27L treated cells. Intriguingly, treatment with HERA-CD27L also upregulated the expression of other co-stimulatory members of the TNFRSF, including CD357 (GITR), CD134 (OX40), and CD137 (4-1BB), which suggests a possible additive role for other TNFRSF agonists in the prolongation of the immune response.

In addition to showing the superiority of the hexavalent HERA-CD27L over trimeric CD27L, these *in vitro* studies also showed that treatment with HERA-CD27L alone did not produce any response in unstimulated T cells, suggesting that a TCR-mediated, and therefore antigen-specific, signal is required for the HERA-CD27L-mediated enhancement of T cell activity. This is clearly an advantage compared to other non-specific strategies, such as checkpoint inhibition or cell depletion-based therapies, that lead to serious immune-related adverse events (irAEs) due to their broad mechanisms of action (22–24).

Although no proliferative/differentiative responses were visible following HERA-CD27L treatment of unstimulated T cells, an almost complete loss of surface CD27 expression was observed. In contrast, T cells simultaneously treated with anti-CD3 antibody and HERA-CD27L had transient and only slightly reduced expression of CD27. It has been shown that CD27 is shed from cells following ligation (25, 26). It is also clear that activated T cells are able to maintain expression of CD27. These data suggest that synchronization of the antigen-specific TCR signal and the CD27 agonistic signal could be required for optimum activity. Therefore, chronic or sustained anti-CD27 activity could lead to desensitization of T cells and the long serum half-life of agonistic antibodies might be especially disadvantageous in this regard. In fact, it has been suggested that some tumors have developed a defense mechanism based on stimulation of CD27 shedding from tumor-infiltrating T cells (8, 27). While the exact mechanisms leading to loss of CD27 expression should be investigated in the future, it is interesting to speculate about the potential consequences of increased concentrations of soluble CD27. The observation of elevated levels of soluble CD27 in both autoimmune diseases as well as cancer highlights the complicated biology of soluble and membrane bound forms of all TNFSF members, such as Fas/FasL and CD40/CD40L, as well as the specific complexity of CD27 (28, 29). The most direct consequence could be for soluble CD27 to bind/sequester HERA-CD27L and therefore, limit efficacy. Soluble CD27 has been measured in the serum of healthy volunteers and various patient groups in the picogram to low nanogram per milliliter range (28, 29). At the doses reported here, a maximum serum concentration of HERA-CD27L in mice and cynomolgus monkeys was measured in the microgram per milliliter range. Together with the *in vivo* activity data, HERA-CD27L seems to be present in sufficient amount to activate T cells.

The superiority and safety of the hexavalent HERA-CD27L was also demonstrated *in vivo* using an antigen-specific adoptive transfer model. Antigen-specific T cells underwent significantly more clonal expansion following antigen challenge and treatment with HERA-CD27L compared to the trimeric CD27L. Since most T cells express CD27, there is potential for non-specific and bystander T cell activation (for example, self-antigen-specific T cells). These potential dangers were examined by comparing OVA-specific and non-specific T cells in the same environment. Even in the environment where 30–40% of all CD8+ T cells are responding to their specific antigen, the non-specific CD8+ T cells do not show any signs of activation. This system, which mimics a more realistic T cell response, also demonstrated the specificity, and therefore safety, of HERA-CD27L as the endogenous T cells were not affected by treatment. Having a requirement for a TCR-dependent and antigen-specific signal as well as having a clearly defined agonistic forward-signaling mechanism of action means that irAEs can be minimized with HERA-CD27L.

The potent anti-tumor efficacy of the hexavalent HERA-CD27L was demonstrated in two different syngeneic mouse tumor models, MC38-CEA and CT26wt. Significant TGI was observed in both tumor models when HERA-CD27L was used as a single agent. In the CT26wt model, HERA-CD27L also showed superior activity compared to anti-PD-1 antibody. Furthermore, the combination of HERA-CD27L and anti-PD-1 antibody showed additive effects on TGI highlighting the importance of both T cell activation and checkpoint inhibition in anti-tumor immunity. This suggests that pro-stimulatory and anti-inhibitory strategies should be employed in combination in order to achieve the best clinical results.

It is becoming increasingly clear that TNFRSF receptors play an important role in activating the immune system and, consequently, initiating and prolonging anti-tumor immunity. This has resulted in the development of multiple distinct approaches to create TNFRSF receptor agonists. Induction of CD27 signaling can be broadly accomplished with its natural ligand (CD70/CD27L) or with agonistic antibodies. As we have demonstrated here, the hexavalent HERA-CD27L induced potent T cell activation and anti-tumor immunity. In contrast, the trimeric CD27L was significantly less effective, presumably due to its inability to properly cluster CD27. Furthermore, treatment with the bivalent clinical benchmark antibody significantly reduced the proliferation of stimulated T cells. In addition, this benchmark antibody failed to induce CD27-mediated signaling activity at a wide range of concentrations. These results support the concept that bivalent compounds, like antibodies, have even more trouble clustering a sufficient number of receptors to transmit a productive signal (8, 10, 13, 14, 22, 30). The data also support our previous findings with HERA-TRAIL suggesting that TNFRSF receptor clustering, best achieved by higher order structures based on the natural ligand, is required for full induction of down-stream signaling and function (14). These results also provide evidence that these bivalent antibodies can interfere with the anti-tumor immune response by blocking the natural CD27/CD70 signaling pathway. In fact, it has been well documented that this clinical benchmark antibody blocks binding of the natural ligand CD70 to CD27 (15).

These results and others have also shown that the actual “agonistic” activity of antibodies targeting TNFRSF receptors, including this benchmark anti-CD27 antibody, is fully dependent on additional crosslinking via the Fc domain of the antibodies (13, 30–33). This crosslinking occurs through interactions between the antibody Fc domain and FcγR on the surface of innate immune cells, such as macrophages, DCs and NK cells, and B cells. These interactions do provide limited forward-signaling agonist activity, unfortunately, Fc/FcγR interactions also result in unnecessary backward-signaling events that can have detrimental consequences (32). In fact, Fc/FcγR interactions have been shown to be responsible for many of the irAEs elicited by antibody-based strategies. Depending on the FcγR-expressing cell and local microenvironment, Fc/FcγR interactions can have a broad array of effects on the immune system, ranging from stimulatory to even inhibitory consequences. The most commonly associated Fc/FcγR-mediated effector functions include antibody-dependent cell-mediated cytotoxicity (ADCC), antibody-dependent cell-mediated phagocytosis (ADCP) and antibody-dependent cytokine release (ADCR) (22, 34, 35). Through ADCC and ADCP, FcγR binding leads to the elimination of CD27 expressing T cells, an unnecessary side effect associated with antibody-based therapy. In fact, reduced numbers of CD4+ T cells and Treg cells were observed in both pre-clinical and Phase I clinical trials of this benchmark anti-CD27 antibody (10, 36). While the reduction in effector T cell numbers suggests further limitations of efficacy, the elimination of Treg cells is especially problematic as there are many dangers associated with Treg cell depletion as a therapeutic strategy (37). The most obvious relates to the control that Treg cells normally have over the large pool of self-reactive T cells (38, 39). The non-specific depletion of Treg cells can unleash these self-reactive T cells and lead to even more irAEs as has been seen with anti-CTLA-4 antibodies. FcγR-mediated backward-signaling can also result in ADCR, or cytokine release syndrome, where innate immune cell activation leads to the systemic production of pro-inflammatory cytokines (22, 40). This serious condition has already led to the early termination of multiple antibody-based clinical trials. Since HERA-CD27L has a non-functional FcγR binding domain, HERA-CD27L will have a vastly superior safety profile compared to antibody-based approaches.

In addition to FcγR-mediated irAEs, the long half-life of antibodies, in comparison to HERA-CD27L, can also lead to serious complications because the stimulation will be present for an extended period of time. This is especially important for CD27 where sustained or chronic CD27 signaling has been shown to lead to depletion of T cells, B cells and NK cells *in vivo* (19, 41–43). Prolonged exposure to anti-CD27 antibodies will also result in continuous shedding of CD27. These consequences of CD27 overstimulation, including depletion of effector cells, CD27 desensitization due to shedding and potential pro-survival effects on Treg cells, have prompted many to suggest that timing and dosing of CD27 stimulation will be critically important (8, 10, 19). Unlike inhibitory or checkpoint-blocking immunotherapies that depend on long half-life and steric hindrance, classic advantages of antibodies, to continuously prevent specific receptor/ligand interactions, immuno-stimulatory strategies targeting TNFRSF receptors need to have the correct exposure and structure to generate a productive and beneficial signal. Stimulatory strategies only need to be long enough to boost the immune response without being too long to cause problems due to chronic stimulation or overstimulation. The short half-life of HERA-CD27L makes it the ideal candidate for exploring this area.

Various strategies have been proposed for targeting CD27 for cancer therapy. As we have shown here, the hexavalent HERA-CD27L has superior activity *in vitro* and *in vivo* compared to a trimeric CD27L and a bivalent clinical benchmark antibody. We have also described how antibody-based approaches have limited efficacy and increased risk for development of irAEs due to their long serum half-life, Treg cell depletion and dependence on FcγR binding. This is especially important given that all newly developed immuno-oncology compounds will probably be used in combination with checkpoint inhibitors that already have high propensity for irAE development (22, 24). In summary, herein we describe the development of HERA-CD27L, a true CD27 agonist with a clearly defined forward-signaling mechanism of action and independent of FcγR-based mechanisms. HERA-CD27L is perfectly suitable for standard large-scale production processes and significantly enhances anti-tumor immunity.

## Author contributions

All authors were involved in the conception, design and development of methodology for this study. Acquisition, analysis and interpretation of data was also accomplished by all authors. MT, DR, and OH were responsible for writing the manuscript. All authors contributed to drafting, revising, and approving the final article.

### Conflict of interest statement

All authors are current or former employees of Apogenix AG. The reviewer LD and the handling Editor declared their shared affiliation.
