# Supplementary material for: A Single-Chain-Based Hexavalent CD27 Agonist Enhances T Cell Activation and Induces Anti-Tumor Immunity
Source: Front Oncol. 2018 Sep 19;8:387. doi: 10.3389/fonc.2018.00387 (PMC6160747; doi:10.3389/fonc.2018.00387)
Supplement: Supplementary file 1 [file Presentation_1.pdf]

Supplementary Material

Title. A single-chain-based hexavalent CD27 agonist enhances T cell activation and induces anti-tumor immunity

Authors

Meinolf Thiemann, David M. Richards, Karl Heinonen, Michael Kluge, Viola Marschall, Christian Merz, Mauricio Redondo-Müller, Tim Schnyder, Julian P. Sefrin, Jaromir Sykora, Harald Fricke, Christian Gieffers and Oliver Hill*

*Corresponding author

Dr. Oliver Hill, VP Molecular Biology / Protein Engineering

Email: oliver.hill@apogenix.com

Supplementary Figures and Tables

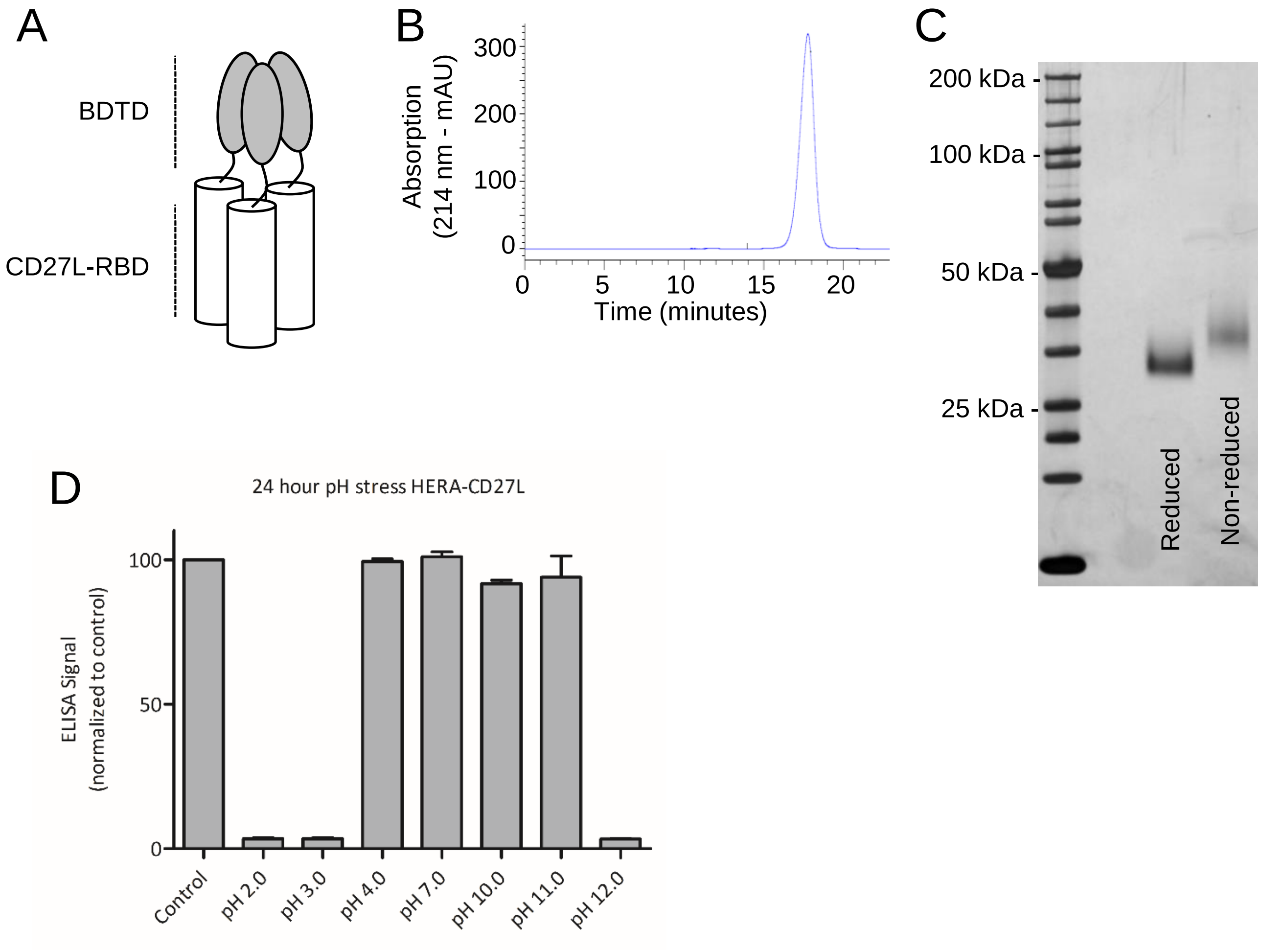

SUPPLEMENTARY FIGURE S1. Structure, design and production of a trimeric CD27L and pH stability of HERA-CD27L. **(A)** Schematic depiction of the structure of the trimeric CD27L. For engineering of the trimeric CD27L, one CD27L (CD70) protomer sub-sequence (called the CD27L-receptor binding domain or CD27L-RBD) was fused to a bacteriophage-derived trimerization domain (BDTD). **(B)** Purification was accomplished by a two-step process combining AFC followed by preparative SEC. For analytical SEC, purified trimeric CD27L was detected by online measurement of absorption at 214 nm. Content of monomer and aggregates was calculated as the AUC from the elution profile of the SEC. Trimeric CD27L eluted as a single peak and showed no detectable aggregates. **(C)** Purity and aggregation status of purified trimeric CD27L was also assessed by non-reducing and reducing SDS-PAGE. **(D)** pH stability was assessed by exposing purified HERA-CD27L to various pH buffers from pH 2.0 to pH 12.0. 24 hours after re-buffering, aliquots were taken and frozen at <- 65°C prior to measuring ELISA binding to immobilized human CD27-Fc. Values are mean ± S.D. OD at a wavelength of 450 nm (with a 630 nm correction) normalized to binding of untreated HERA-CD27L. Representative data from at least three independent experiments are shown.

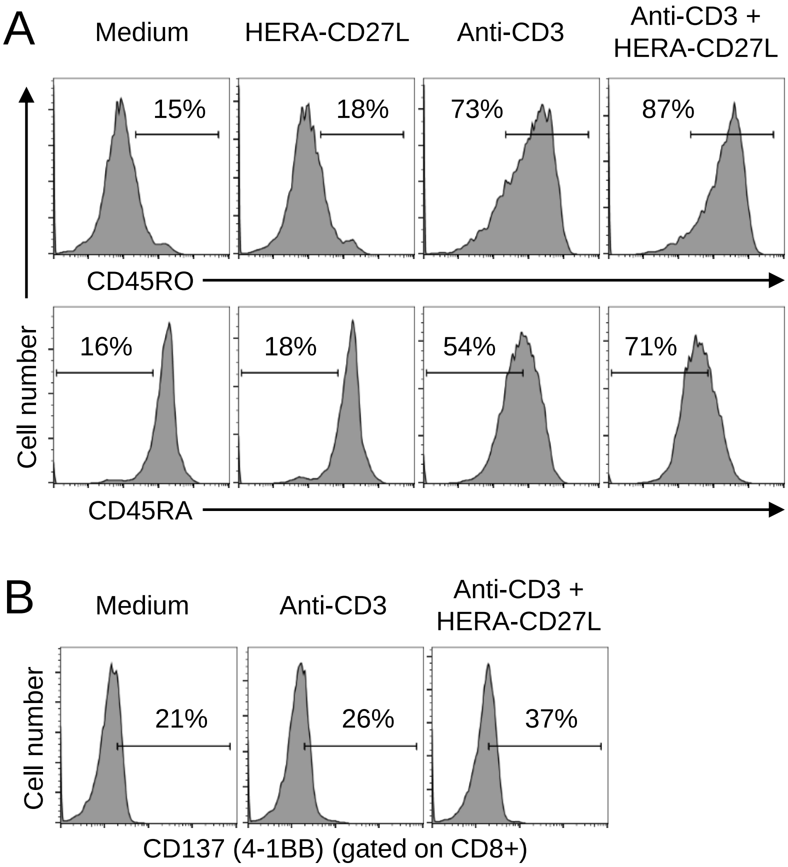

SUPPLEMENTARY FIGURE S2. HERA-CD27L enhances T cell differentiation following stimulation *in vitro*. **(A)** Naïve CD4+ T cells were isolated from the peripheral blood of healthy volunteers and stimulated with anti-CD3 antibody or medium control in the presence of HERA-CD27L (100 ng/mL) or vehicle control (PBS), as indicated. On day five, T cells were harvested, stained for surface expression of CD45RO and CD45RA and examined by flow cytometry. **(B)** Naïve CD8+ T cells were stimulated with anti-CD3 antibody in the presence of HERA-CD27L or vehicle control (PBS), as indicated. On day four, T cells were harvested, stained for surface expression of CD137 (4-1BB) and examined by flow cytometry. Numbers indicate the percentage of cells within the defined region. Representative histograms, gated on live single cells, from the median sample of triplicates from at least three independent experiments are shown.

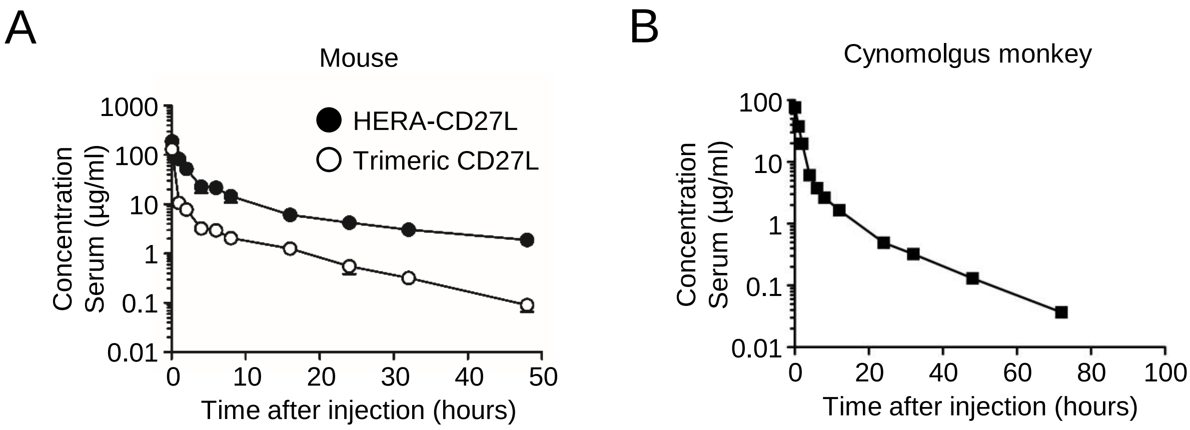

SUPPLEMENTARY FIGURE S3. Pharmacokinetics of HERA-CD27L and trimeric CD27L in mouse and cynomolgus monkey. **(A)** Female CD1 mice were administered with 10 mg/kg b.w. of HERA-CD27L or trimeric CD27L as a single i.v. injection and whole blood was collected after test item administration. Serum was prepared and HERA-CD27L or Trimeric CD27L serum concentration was quantitated by ELISA assays assessing functional binding of HERA-CD27L to human CD27-Fc. **(B)** Male cynomolgus monkeys were administered with 3 mg/kg b.w. of HERA-CD27L as a single i.v. injection and whole blood was collected after test item administration. Serum was prepared and HERA-CD27L serum concentration was quantitated by ELISA assays assessing functional binding of HERA-CD27L to human CD27-Fc.

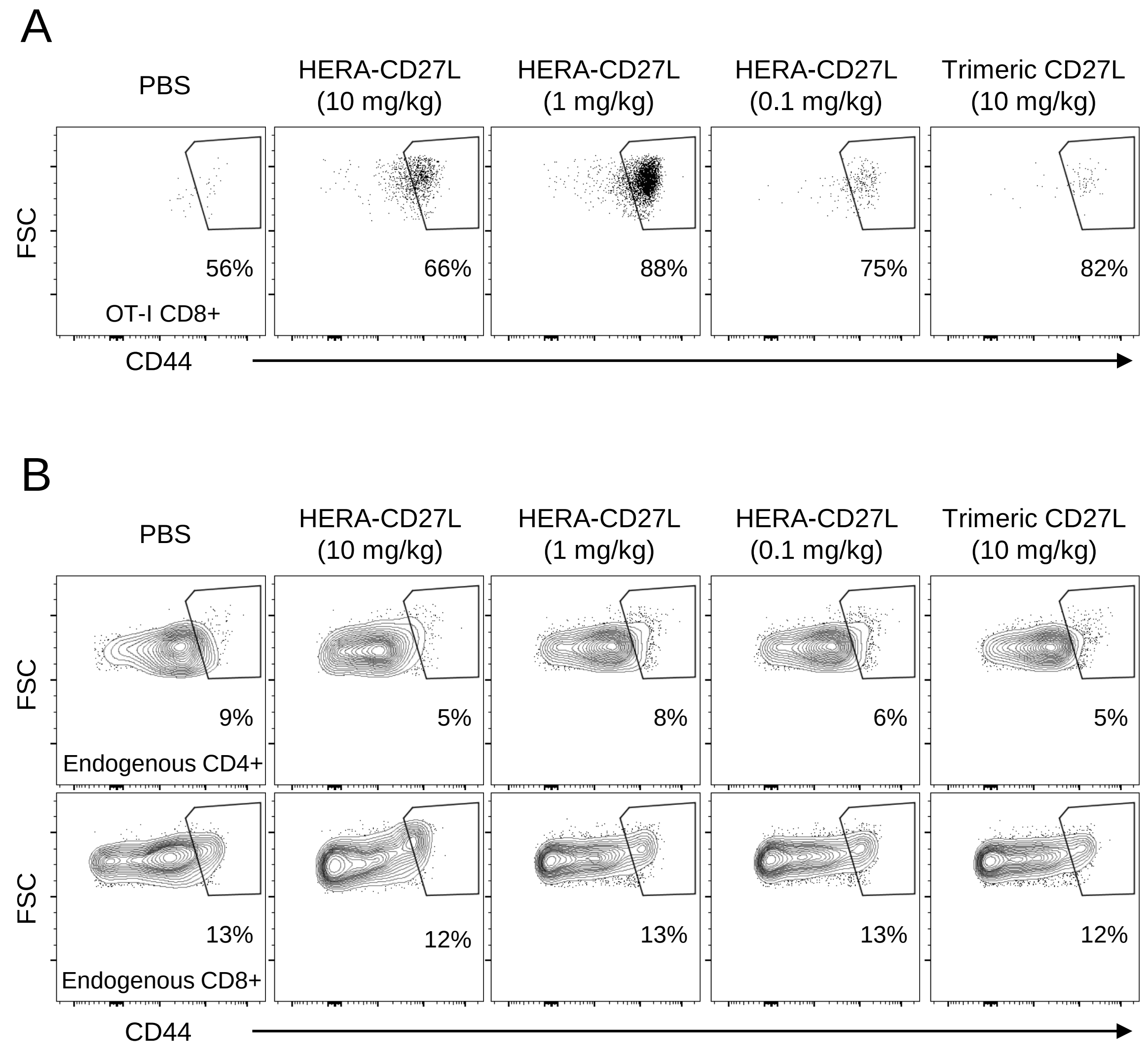

SUPPLEMENTARY FIGURE S4. HERA-CD27L significantly boosts the antigen-specific CD8+ T cell response *in vivo* without affecting endogenous T cells. **(A, B)** Female C57Bl/6 recipient mice were adoptively transferred with 2 x 10^6 CD8+ OT-I T cells, challenged with OVA protein and treated with a single injection of HERA-CD27L (0.1, 1 and 10 mg/kg b.w.), trimeric CD27L (10 mg/kg b.w.) or vehicle control (PBS). Serial blood samples were obtained from each animal over a two-week period and on day six, cells were stained for surface expression of CD44 and examined by flow cytometry. **(A)** OT-I T cells were identified by flow cytometry using CD8 and a K^b/OVA tetramer. Dot plots are used to demonstrate the number of total OT-I cells recovered in each group. **(B)** Endogenous CD4+ T cells and endogenous CD8+ T cells were identified using CD4 or CD8 and were tetramer negative. Numbers indicate the percentage of cells (CD44high) within the gated region. Representative contour plots, gated on live single T cells, from the median sample of triplicates from at least three independent experiments are shown. One-way ANOVA plus post hoc Bonferroni multiple comparisons analysis was conducted on all figures and showed no significant differences.

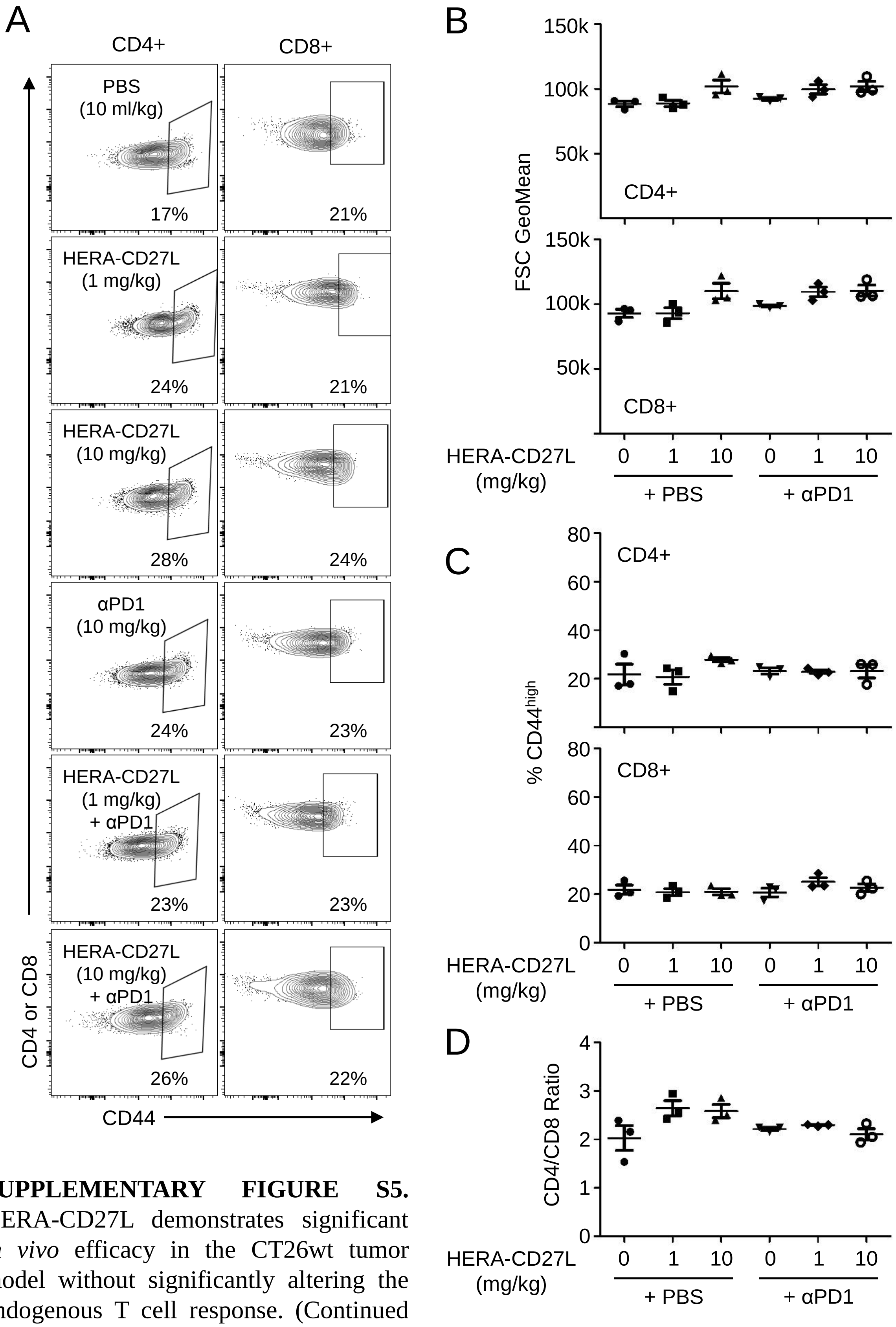

SUPPLEMENTARY FIGURE S5. HERA-CD27L demonstrates significant *in vivo* efficacy in the CT26wt tumor model without significantly altering the endogenous T cell response. (Continued next page).

SUPPLEMENTARY FIGURE S5. HERA-CD27L demonstrates significant *in vivo* efficacy in the CT26wt tumor model without significantly altering the endogenous T cell response. **(A – D)** Freshly cultured CT26wt tumor cells (5 x 10^5 in 100 µl RPMI) were implanted s.c. into the right flank of 6-week-old female BALB/c mice. Tumor volume was determined twice weekly by caliper measurement. Mice were randomized on day 11 into groups of 12 mice per treatment group with a mean primary tumor volume of 83 mm^3. All animals were treated i.v. three times, indicated by the black triangles. One to four animals in each group were terminated early due to ethical considerations that were independent of tumor size. The in-life phase of the study finished on day 25 following tumor implantation. On day 25, spleens were harvested, stained for surface marker expression and examined by flow cytometry. **(A)** Representative contour plots, gated on live single CD4+ or CD8+ T cells, left and right columns, respectively, are shown. **(B – D)** Horizontal lines indicate the mean (n = 3) ± S.D for each group. **(B)** The forward scatter (FSC) geometric mean is shown for CD4+ or CD8+ T cells, top and bottom rows, respectively. **(C)** The percentage of $CD44^{high}$ cells is shown for CD4+ or CD8+ T cells, top and bottom rows, respectively. **(D)** The ratio of CD4+ to CD8+ T cells is shown. One-way ANOVA plus post hoc Bonferroni multiple comparisons analysis was conducted on all figures and showed no significant differences.

SUPPLEMENTARY TABLE S1. Summary of HERA-CD27L stability under various conditions.

Stability	Condition	Results
Storage stability (up to 2 weeks)	5°C	2 weeks
	RT	2 weeks
	37°C	4 days
Freeze/thaw stability (up to 5 cycles)		5 cycles
Heat stress (10 min)		50°C
pH stability	30 min	pH 4.0-11.0
	1 hour	pH 4.0-11.0
	24 hours	pH 4.0-11.0

SUPPLEMENTARY TABLE S2. Summary of the binding constants of HERA-CD27L and trimeric CD27L to human (hs) and mouse (mm) CD27-Fc.

Receptor	Ligand	K_D
hsCD27-Fc	HERA-CD27L	2 nM
hsCD27-Fc	trimeric CD27L	4 nM
mmCD27-Fc	HERA-CD27L	22 pM
mmCD27-Fc	trimeric CD27L	6 nM

SUPPLEMENTARY TABLE S3. Summary of the pharmacokinetic properties of HERA-CD27L and trimeric CD27L in mouse and cynomolgus monkey.

Ligand	Species	Dosing [mg/kg b.w.]	$AUC_{0\text{-}inf}$ [µg · h/ml]	$t_{1/2}$ [h]
trimeric CD27L	CD-1 mice	10	134	8.5
HERA-CD27L	CD-1 mice	10	597	14.5
HERA-CD27L	cynomolgus monkey	3	162	11.4
